# Blood-based biomarkers of age-associated epigenetic changes in human islets associate with insulin secretion and diabetes

**DOI:** 10.1038/ncomms11089

**Published:** 2016-03-31

**Authors:** Karl Bacos, Linn Gillberg, Petr Volkov, Anders H Olsson, Torben Hansen, Oluf Pedersen, Anette Prior Gjesing, Hans Eiberg, Tiinamaija Tuomi, Peter Almgren, Leif Groop, Lena Eliasson, Allan Vaag, Tasnim Dayeh, Charlotte Ling

**Affiliations:** 1 Epigenetics and Diabetes Unit, Department of Clinical Sciences, Lund University Diabetes Centre, CRC 91:12, Jan Waldenströms gata 35, 20502 Malmö, Sweden; 2 Department of Endocrinology, Diabetes and Metabolism, Rigshospitalet, Tagensvej 20, 2200 Copenhagen, Denmark; 3 Faculty of Health Sciences, University of Copenhagen, Blegdamsvej 3, 2200 Copenhagen, Denmark; 4 The Novo Nordisk Foundation Center for Basic Metabolic Research, Section of Metabolic Genetics, University of Copenhagen, Universitetsparken 1, 2100 Copenhagen, Denmark; 5 Department of Cellular and Molecular Medicine, Faculty of Health and Medical Sciences, University of Copenhagen, Blegdamsvej 3, 2200 Copenhagen, Denmark; 6 Department of Endocrinology, Abdominal Centre, Helsinki University Hospital; Folkhalsan Research Center; Research Program for Diabetes and Obesity, Hartmaninkatu 8, Helsinki 00014, Finland; 7 Finnish Institute for Molecular Medicine, University of Helsinki, Hartmaninkatu 8, 00014 Helsinki, Finland; 8 Diabetes and Endocrinology, Department of Clinical Sciences, Lund University Diabetes Centre, Jan Waldenströms gata 35, 20502 Malmö, Sweden; 9 Islet cell exocytosis, Department of Clinical Sciences, Lund University Diabetes Centre, Jan Waldenströms gata 35, 20502 Malmö, Sweden

## Abstract

Aging associates with impaired pancreatic islet function and increased type 2 diabetes (T2D) risk. Here we examine whether age-related epigenetic changes affect human islet function and if blood-based epigenetic biomarkers reflect these changes and associate with future T2D. We analyse DNA methylation genome-wide in islets from 87 non-diabetic donors, aged 26–74 years. Aging associates with increased DNA methylation of 241 sites. These sites cover loci previously associated with T2D, for example, *KLF14*. Blood-based epigenetic biomarkers reflect age-related methylation changes in 83 genes identified in human islets (for example, *KLF14, FHL2, ZNF518B* and *FAM123C*) and some associate with insulin secretion and T2D. DNA methylation correlates with islet expression of multiple genes, including *FHL2*, *ZNF518B, GNPNAT1* and *HLTF.* Silencing these genes in β-cells alter insulin secretion. Together, we demonstrate that blood-based epigenetic biomarkers reflect age-related DNA methylation changes in human islets, and associate with insulin secretion *in vivo* and T2D.

## Abstract (web summary)

Aging is associated with impaired pancreatic islet function, increased risk of type 2 diabetes, and changes in DNA methylation. Here the authors find blood-based biomarkers that reflect age-associated DNA methylation changes in human pancreatic islets associated with insulin secretion and diabetes.

## Graphical abstract

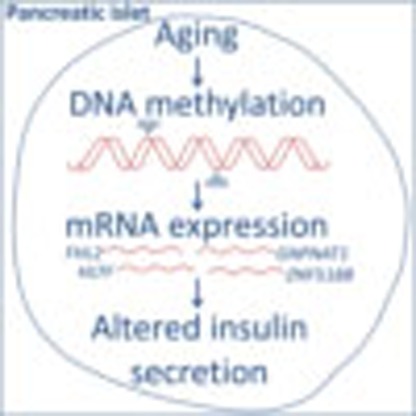

Aging is an important risk factor for type 2 diabetes (T2D)^1^,^2^ and the increasing age of the world's populations contributes to the rapidly growing prevalence of T2D. Elderly people often exhibit insulin resistance and progressive decline in glucose tolerance. Euglycaemia is initially sustained by an adaptive increase in insulin secretion from the pancreatic β-cells. However, aging β-cells may eventually fail to adapt to increasing demands for insulin production, which manifests in hyperglycaemia and T2D^1^. Why aging β-cells fail is unclear, and understanding this pathogenetic process could contribute to understanding the roots of T2D. One possible factor may be that aging causes epigenetic changes that affect gene expression and thereby potentially insulin secretion in pancreatic islets^3^.

Epigenetic alterations, such as changes in DNA methylation, have been linked to several diseases, including T2D^2^,^3^,^4^,^5^,^6^,^7^,^8^. We have previously identified increased DNA methylation and decreased expression of the insulin gene^5^ and *PDX1* (ref. 6), a key transcription factor for β-cell development and function, in pancreatic islets from subjects with T2D. More recently, we analysed DNA methylation genome-wide in islets from diabetic subjects and non-diabetic controls and identified multiple genes with altered methylation and expression in diabetic islets^3^. These epigenetic changes were further associated with perturbed islet hormone secretion, and likely contribute to hyperglycaemia. Epigenetic variation in human islets seems to also contribute to sex differences in insulin secretion^9^. We and others have also identified some age-related DNA methylation changes in human islets, skeletal muscle, adipose tissue, brain and blood cells^3^,^10^,^11^,^12^,^13^,^14^,^15^,^16^,^17^,^18^. Furthermore, epigenetic changes are associated with islet dysfunction and hyperglycaemia in aging rats^19^,^20^. However, it remains unknown if aging is associated with genome-wide changes in DNA methylation of human islets and if these changes have any effect on islet function and development of T2D.

The possibilities for following pancreatic islet health non-invasively are so far largely limited to nutrient-stimulation of hormone secretion, and analysing epigenetic modifications in human islets *in vivo*/non-invasively remains impossible. There is a great translational scientific and public health potential in identifying novel biomarkers in blood that mirror protective or causal disease associated changes in human islets. Such biomarkers could potentially be used to predict and/or to monitor disease progression in T2D^21^.

In this study, we investigate epigenetic modifications and find that age associates with increased DNA methylation in human pancreatic islets. These epigenetic changes correlate with expression of numerous genes and silencing these genes in β-cells alters insulin secretion. Furthermore, we show that age-associated methylation changes in blood reflect the changes seen in islets, suggesting that blood may be used as a marker for islet DNA methylation. Finally, we investigate prospective cohorts and show that DNA methylation of some of these genes associates with future capacity to secrete insulin and T2D risk.

## Results

### Characteristics of human pancreatic islet donors

The characteristics of the 87 non-diabetic human pancreatic islet donors, aged 26–74 years, are shown in Table 1. While aging was associated with elevated HbA1c levels (*P*=0.028 as analysed by linear regression, Supplementary Fig. 1a), there were no significant associations between age and body mass index (BMI) or *in vitro* glucose-stimulated insulin secretion (Supplementary Fig. 1b,c). Nor was age associated with β- or α-cell content in the islets (Supplementary Fig. 1d,e).

### Age-related DNA methylation changes in human islets

**Figure 1:**
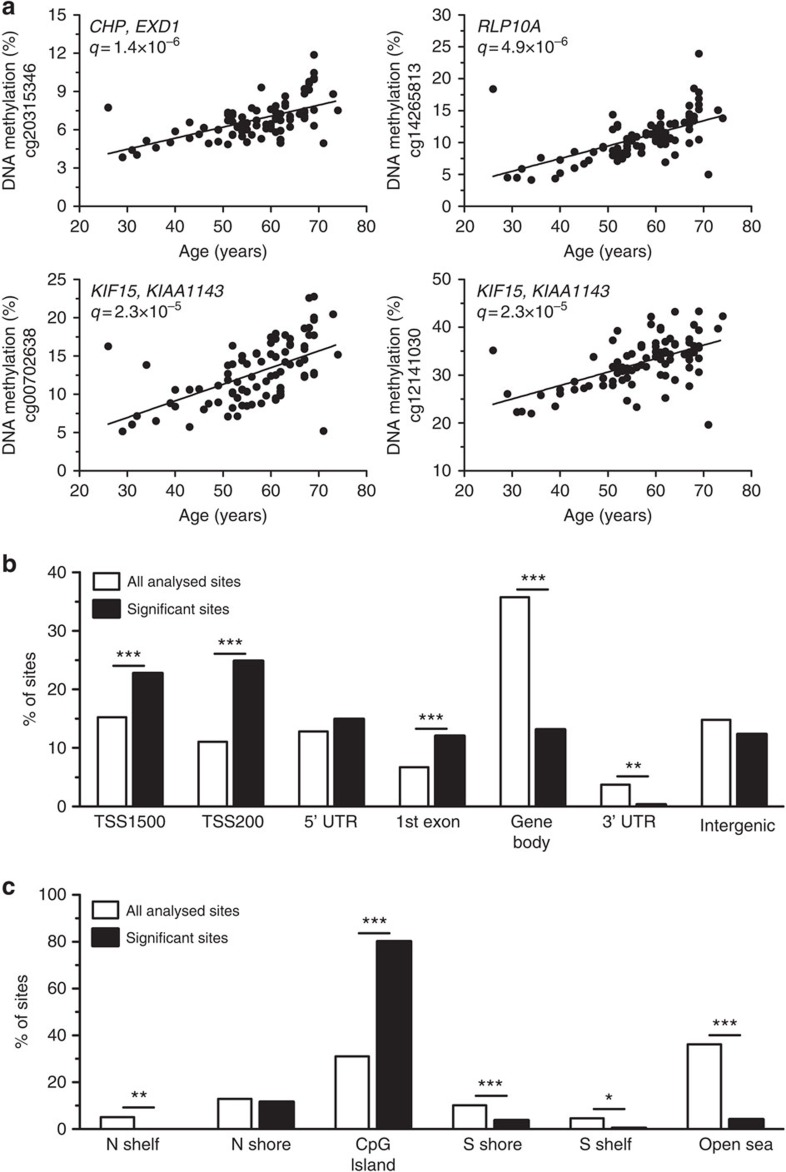
Aging is associated with altered DNA methylation in human pancreatic islets. (**a**) Correlation between age and methylation of four CpG sites with the most significant associations with age in pancreatic islets as analysed by linear regression analysis (*n*=87). (**b**) Distribution of the CpG sites significantly associated with age in relation to gene regions. TSS1500, region 200–1,500 bp upstream of the transcription start site (TSS). TSS200, 200 bp immediately upstream of the TSS. UTR, untranslated region. (**c**) Distribution of the CpG sites significantly associated with age in relation to CpG islands. Northern/Southern shore: 2 kb regions upstream/downstream of the CpG island. Northern/Southern shelf: 2 kb regions outside the shore regions. Open sea: all DNA outside the CpG island regions. *P<0.05, **P<0.01 and ***P<0.001. Significance of the data in **b** and **c** were calculated with a *χ*^2^-test and have been corrected with Bonferroni correction.

To unravel the impact of aging on the human pancreatic islet epigenome, DNA methylation was analysed with the Illumina Infinium HumanMethylation450 BeadChip in islets from the 87 donors. Aging was significantly associated with altered DNA methylation at 241 sites after correction for multiple testing (false discovery rate (FDR)<5%, *q*<0.05, linear regression). These 241 sites were distributed in 154 genes and intergenic regions (Supplementary Data 1). Interestingly, methylation of all significant sites increased with age. The most significant associations between age and DNA methylation in human islets are presented in Fig. 1a. We next evaluated the distribution of the 241 significant methylation sites based on their relation to the nearest genes (Fig. 1b) or CpG islands (Fig. 1c)^22^. The significant sites were enriched in promoter regions (TSS1500 and TSS200), first exon and CpG islands, while they were underrepresented in the gene body, 3′ untranslated region, southern shore, shelf regions and open sea (Fig. 1b,c). None of the probes used to identify significant sites cross-react to other locations in the genome with a perfect match (Supplementary Data 2).

To study the impact of a smaller age difference on methylation in islets, we reanalysed the 241 significant sites from the original analysis in subjects within an age span of the average age±1 s.d. (56.7±10.5 years). Here methylation of 125 sites associated with age (*q*<0.05, linear regression) and methylation of all these increased with age (Supplementary Data 3).

### Functions of genes with age-related methylation changes

**Figure 2:**
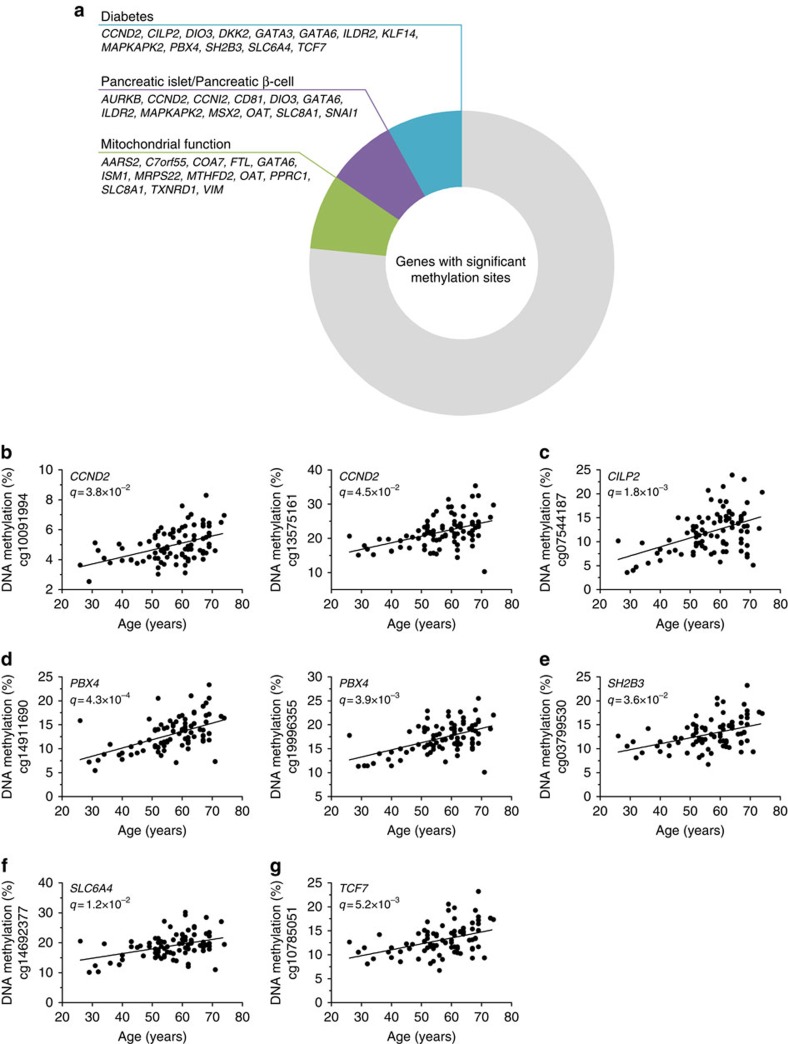
Age-associated methylation changes of genes with importance for diabetes, β-cell function and mitochondria. (**a**) Graphic illustration of the results of a literature study performed on all genes with age-associated methylation changes in human islets. PubMed was searched for papers containing the gene name and each of the following search terms: diabetes, pancreatic islet, pancreatic β-cell or mitochondrial function. Of note, a few genes are included in more than one of the groups. (**b**–**g**) Correlation between age and DNA methylation of CpG sites in genes associated with diabetes risk; *CCND2* (**b**), *CILP2* (**c**), *PBX4* (**d**), *SH2B3* (**e**), *SLC6A4* (**f**) and *TCF7* (**g**) as analysed with linear regression analysis (*n*=87).

To further understand the biological relevance of the identified DNA methylation changes (Supplementary Data 1), we performed a literature search to investigate potential involvement of the 154 differentially methylated genes in islet function and pathogenesis of diabetes. The literature search was performed using each gene name and the following terms; diabetes, pancreatic islet, pancreatic β-cells or mitochondrial function. This showed that 30 (19%) of the identified genes have been associated with diabetes, islet/β-cell function or mitochondrial function, and thus might be important for insulin secretion and glucose homeostasis (Fig. 2a and Supplementary Data 4). These include *CCND2*, *CILP2*, *PBX4, SH2B3, SLC6A4*, *TCF7* and *KLF14* (Fig. 2a–g and Supplementary Data 1 and 4), which have polymorphisms associated with diabetes risk.

### DNA methylation correlates with gene expression in islets

**Figure 3:**
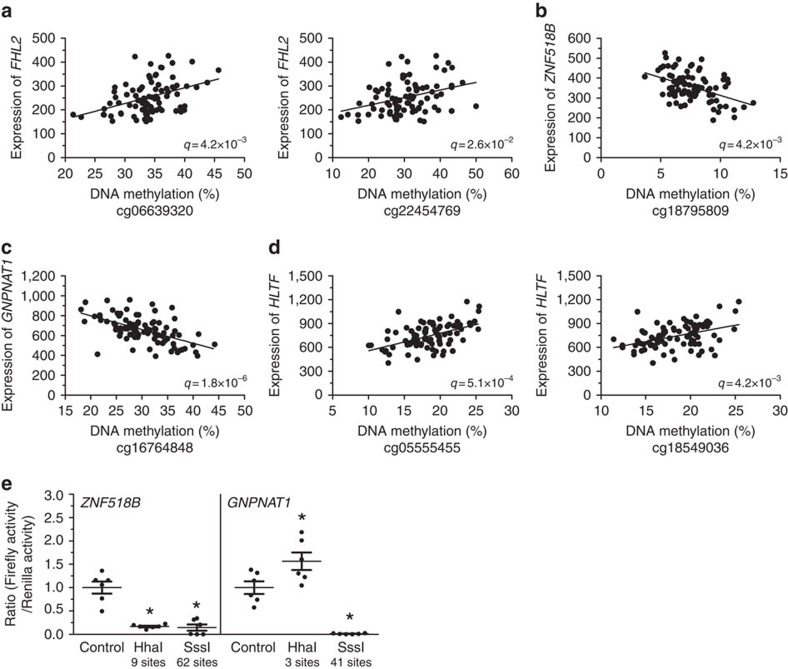
Correlations between DNA methylation and gene expression in human pancreatic islets. DNA methylation of CpG sites in *FHL2* (**a**), *ZNF518B* (**b**), *GNPNAT1* (**c**), and *HLTF* (**d**) and the expression of respective gene correlates significantly in human pancreatic islets (Spearman correlation, *n*=87). *q* values were calculated with the standard Benjamini–Hochberg procedure^70^. (**e**) *ZNF518B* and *GNPNAT1* promoters were tested for their transcriptional activity in luciferase assays after mock-methylation or methylation with HhaI (methylates the internal CpG site in GCGC sequences) or SssI (methylates all CpG sites). Data are presented as mean±s.e.m. of six experiments with three biological replicates in each. *P<0.05 compared to control as analysed by a Wilcoxon signed rank test.

Since DNA methylation may regulate gene expression^23^, we tested whether the identified age-associated methylation changes correlate with expression of respective nearby annotated gene(s), including a total of 154 unique genes. Here methylation of 32 sites correlated with expression of respective gene with *P*<0.05 and 11 of these with *q*<0.05 (Spearman's rank test, Supplementary Data 5). These include *FHL2*, *ZNF518B, GNPNAT1* and *HLTF* (Fig. 3). *ZNF518B* and *GNPNAT1* showed inverse correlations between DNA methylation and expression (Fig. 3b,c). Indeed, methylation in gene promoters can result in diminished expression by inhibiting the binding of transcription factors or recruiting methyl CpG-binding proteins together with HDACs and co-repressors^2^,^23^. However, expression of *FHL2* and *HLTF* correlated positively with the methylation of CpG sites in respective gene, despite the sites being situated in promoter regions (Fig. 3a,d). We hypothesised that methylation of these sites prevent binding of repressive transcription factors, so we used LASAGNA-Search 2.0 to analyse which factors bind to stretches of DNA that contain these CpG sites. This showed that the significant CpG sites in both *FHL2* and *HLTF* promoters are situated within binding sites for repressive transcription factors (Supplementary Data 6). Thus, increased methylation may reverse repression of *FHL2* and *HLTF* and thereby enhance expression.

We next used luciferase assays to study the impact of altered DNA methylation in transcriptional regulation of genes presented in Supplementary Data 5. For this experiment, we selected *ZNF518B* and *GNPNAT1*, two genes where the age-associated methylation changes take place in promoter regions and with inverse correlations between methylation and expression (Fig. 3b,c and Supplementary Data 5). Promoter sequences for *ZNF518B* and *GNPNAT1* were inserted into a luciferase expression plasmid and each construct was mock-methylated or methylated with two different methyltransferases, HhaI and SssI. The number of CpG sites that may be methylated in each respective construct is shown in Fig. 3e. While SssI methylation suppressed reporter expression for both genes, HhaI methylation only suppressed reporter expression for *ZNF518B* (Fig. 3e). Interestingly, methylation of the three GCGC sites in the *GNPNAT1* promoter by HhaI led to increased reporter expression (Fig. 3e). LASAGNA-Search 2.0 analysis showed that two of these GCGC sites are situated in a binding site for the well-known repressor NRSF^24^,^25^ (Supplementary Data 7), possibly explaining this increased reporter expression.

### Differentially methylated genes affect insulin secretion

**Figure 4:**
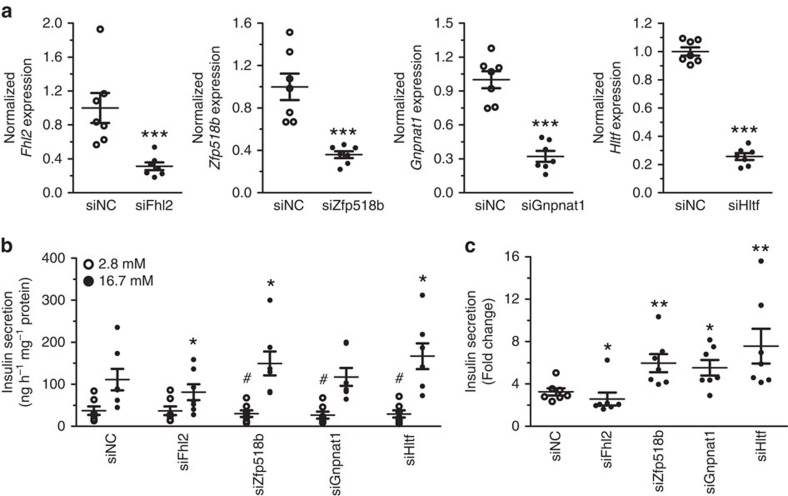
Knockdown of *Fhl2*, *Zfp518b, Gnpnat1* and *Hltf* affects insulin secretion in clonal β-cells. (**a**) Quantification of siRNA-mediated knockdown of *Fhl2*, *Zfp518b*, *Gnpnat1* and *Hltf* in clonal β-cells. Data are presented as the mean±s.e.m. of seven experiments and were analysed with a Mann–Whitney test. ***P<0.001 versus negative control siRNA (siNC). (**b**) Insulin secretion at basal (2.8 mM) and/or stimulatory (16.7 mM) glucose levels were altered in β-cells deficient for *Fhl2*, *Zfp518b*, *Gnpnat1* or *Hltf*. Data are presented as the mean±s.e.m. of seven experiments with three biological replicates in each and were analysed with a Wilcoxon signed rank test. *P<0.05 versus siNC 16.7 mM and ^#^*P*<0.05 versus siNC 2.8 mM. (**c**) The fold change of insulin secretion (the ratio of secretion at 16.7 mM and 2.8 mM glucose) was reduced in β-cells deficient for *Fhl2*, while it was increased in cells deficient for *Zfp518b, Gnpnat1* or *Hltf*. Data are presented as mean±s.e.m. of seven experiments and were analysed with a Mann–Whitney test. *P<0.05 versus siNC and **P<0.01 versus siNC.

We next investigated whether genes with age-associated changes in DNA methylation, and a significant correlation between methylation and expression, affect insulin secretion in β-cells. *FHL2*, *ZNF518B*, *GNPNAT1* and *HLTF,* four of the most significant genes presented in Supplementary Data 5 and Fig. 3, were selected for functional follow-up experiments. We silenced the expression of *Fhl2*, *Zfp518b* (rat homologue of *ZNF518B*), *Gnpnat1* and *Hltf* in clonal rat β-cells using short interfering RNA (*siRNA)*. This reduced expression of respective mRNA by ∼65–75% (Fig. 4a). We then measured insulin secretion at basal (2.8 mM) and stimulatory (16.7 mM) glucose levels in siRNA-transfected β-cells. While silencing of *Fhl2* resulted in diminished insulin secretion at 16.7 mM glucose, *Gnpnat1* deficiency reduced insulin secretion at 2.8 mM glucose (Fig. 4b). In addition, β-cells deficient for *Zfp518b* or *Hltf* expression exhibited reduced insulin secretion at 2.8 mM glucose and increased secretion at 16.7 mM glucose (Fig. 4b). These changes also resulted in altered fold change of insulin secretion (secretion at stimulatory glucose divided by secretion at basal glucose levels) in β-cells deficient for *Fhl2*, *Zfp518b, Gnpnat1* or *Hltf* (Fig. 4c). In summary, silencing of genes with age-related alteration in DNA methylation and associated expression changes have functional effects on clonal β-cells.

### Blood-based biomarkers of islet DNA methylation and function

**Figure 5:**
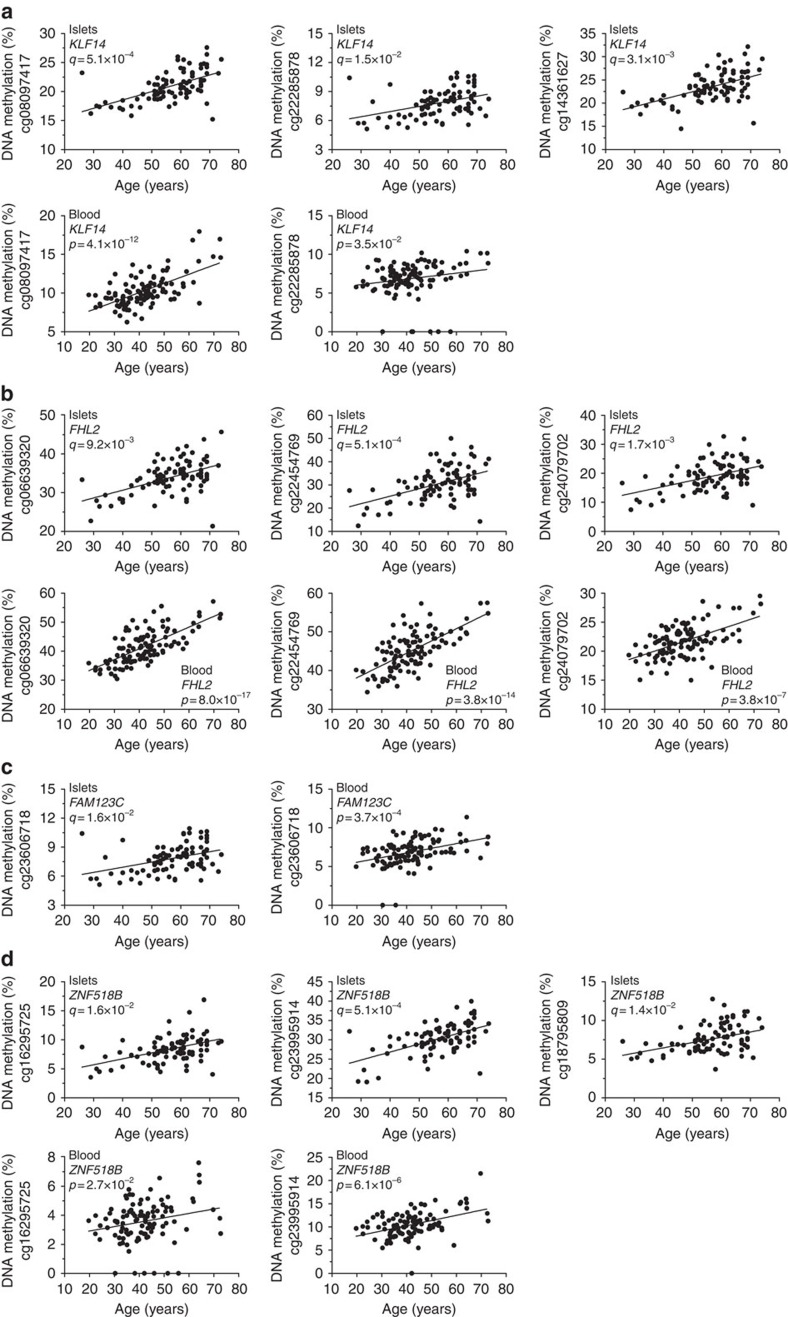
Methylation changes of *KLF14*, *FHL2*, *FAM123C* and *ZNF518B* in human islets are reflected by methylation changes in blood. Association between age and DNA methylation of sites in *KLF14* (**a**), *FHL2* (**b**), *FAM123C* (**c**) and *ZNF518B* (**d**) in pancreatic islets from the human islet cohort (*n*=87, upper panels) and blood from the Danish Family Study at baseline (*n*=112, lower panels), as analysed by linear regression analysis.

Epigenetic biomarkers in blood have more clinical potential than those in pancreatic islets as islets cannot be non-invasively assessed, so we tested whether age-related DNA methylation changes in blood cells reflect the epigenetic changes identified in human pancreatic islets and if they may be used to monitor insulin secretion *in vivo*. We first compared our significant results from human islets with data from a previously published study, which examined the impact of age on DNA methylation in leucocytes from a large human cohort; 421 individuals aged 14–94 years^15^. Intriguingly, 139 (57.7%) of the 241 methylation sites that significantly associated with age in pancreatic islets did so also in leucocytes (Supplementary Data 8). The methylation level of all overlapping sites increased with age in both islets and leucocytes. Notably, methylation of numerous of these sites has been reported to associate with age in blood in additional studies^10^,^12^,^13^ (Supplementary Data 8). The 139 overlapping sites cover 83 unique genes, including *KLF14*, *FHL2*, *FAM123C* and *ZNF518B*. Depictions of significant associations between age and DNA methylation for CpG sites in these four genes in human pancreatic islets are presented in Fig. 5a–d.

We next used pyrosequencing to experimentally test if we also could identify these age-related changes in DNA methylation in blood. However, since we did not have access to blood samples from the islet donors, we analysed DNA methylation of *KLF14*, *FHL2*, *FAM123C* and *ZNF518B* in blood samples taken from another cohort of 112 individuals from the Danish Family Study^26^,^27^. We chose these genes as their methylation levels previously have been found to associate with age in blood (all four genes, Supplementary Data 8), they showed functional effects in β-cells (*FHL2* and *ZNF518B*, Fig. 4) or have been associated with T2D risk (*KLF14* (refs 28, 29)). We also analysed DNA methylation in blood of *GNPNAT1* and *HLTF*, which showed age-associated methylation changes in islets (Supplementary Data 1 and Supplementary Fig. 2, left panels) and had functional effects in β-cells (Fig. 4), but age-associated methylation changes in blood have not been described for the studied sites in these two genes. In the Danish Family Study, blood samples were available from two time points separated by ∼10 years and the individuals had a wide range in age (20–73 years at baseline and 32–83 years at follow-up, Supplementary Data 9). Importantly, increased age was significantly associated with increased methylation of *KLF14*, *FHL2*, *FAM123C* and *ZNF518B,* in the Danish Family Study at both baseline and follow-up (Fig. 5a–d and Supplementary Fig. 3). For *GNPNAT1* and *HLTF*, however, the associations were negative or non-significant, respectively (Supplementary Fig. 2). Also, the degree of DNA methylation increased for *KLF14*, *FHL2*, *FAM123C* and *ZNF518B* from baseline to follow-up (Supplementary Fig. 4) showing an intra-individual increase in methylation during aging.

We next used data from the study by Slieker *et al.*^30^ to compare the degree of methylation of the studied sites in *KLF14*, *FHL2*, *FAM123C* and *ZNF518B* in blood and pancreas taken from the same human donors. Most of the sites exhibit similar methylation levels in blood and pancreas, and the methylation data in the two tissues correlated significantly (*P*=4.2 × 10^−11^ as analysed by Spearman's rank test, Supplementary Fig. 5). Together, our data and the data from the study by Slieker *et al.*^30^ support that age-related methylation changes found in blood in many cases are similar to methylation changes in pancreatic islets and potentially can be used as biomarkers for alterations in primary tissues for diabetes.

Moreover, the pyrosequencing assays used for analysis of *KLF14*, *FHL2, FAM123C, ZNF518B, GNPNAT1* and *HLTF* all cover several methylation sites not included on the methylation array. These additional methylation sites were in most cases also significantly associated with age at both baseline and follow-up (Supplementary Figs 6 and 7), indicating that the sites identified by the array lie within genomic regions that are increasingly methylated during aging. It should further be noted that the degree of baseline and follow-up DNA methylation for all analysed sites (except cg22285878 in *KLF14*) correlated significantly for respective gene (median *P* value 8.8 × 10^−11^ and 1.6 × 10^−10^, respectively, as analysed by Spearman's rank test).

It is possible that age-associated alterations in blood cell composition affect our results^31^,^32^. To investigate this, we used data for the studied CpG sites in *KLF14*, *FHL2, FAM123C, ZNF518B* and *GNPNAT1* from the study by Reinius *et al.*^33^, where they analysed DNA methylation in isolated blood cell populations. There were small differences in DNA methylation between lymphoid and myeloid cell populations for some of these sites (Supplementary Fig. 8). However, several of these differences actually strengthen our results as the methylation level is higher in lymphoid cells than in myeloid cells and aging is associated with a decline in lymphoid cell numbers^31^,^32^.

In the Danish Family Study, age was significantly associated with elevated levels of both insulin and C-peptide during an oral glucose tolerance test (OGTT) at follow-up (Table 2). Also HbA1c and glucose levels associated positively with age in these individuals (Table 2), which is in agreement with what we found in our islet donors (Supplementary Fig. 1). Moreover, age was negatively associated with disposition index (DI), a measure of insulin secretion corrected for insulin sensitivity (Table 2). We proceeded to test if DNA methylation of CpG sites in genes with age-associated methylation changes identified in both pancreatic islets and blood (*KLF14*, *FHL2*, *FAM123C, ZNF518B* and *GNPNAT1*) associated with measures of insulin secretion in the Danish Family Study, independent of age, and therefore can be used as biomarkers for β-cell function. We found that methylation at baseline of CpG sites in *KLF14* and *ZNF518B* associated with elevated insulin and C-peptide levels measured during an OGTT at the same time point (associations with *P*<0.05 as analysed by linear mixed effect models are presented in Table 3). We next tested if DNA methylation of *KLF14*, *FHL2*, *FAM123C*, *ZNF518B* and *GNPNAT1* measured in blood at baseline associates with insulin secretion measured at follow-up in the Danish Family Study. Indeed, DNA methylation in *KLF14* and *ZNF518B* at baseline was significantly associated with altered insulin levels measured during an OGTT at follow-up after 10.4±0.9 years (associations with *P*<0.05 as analysed by linear mixed effect models are presented in Table 4). Together, these data suggest that epigenetic markers in blood associate with measures of insulin secretion *in vivo* and may be used to predict future capacity to secrete insulin.

### DNA methylation associates with future T2D

To investigate if epigenetic markers associate with risk of future diabetes, we used pyrosequencing to analyse DNA methylation of *KLF14, FHL2, FAM123C*, *ZNF518B* and *GNPNAT1* in blood from subjects in the Botnia prospective study^34^ (Supplementary Data 10). These participants were healthy at baseline and were followed prospectively with repeated OGTTs to detect progression to T2D (mean follow-up time 10.8 years)^34^. Higher DNA methylation of CpG sites in *KLF14*, *FHL2* and *GNPNAT1* was associated with smaller hazard ratios and hence a lower risk of future T2D (Table 5). The relative risk per 1% increase in methylation was 0.88 for a site in *KLF14*, 0.94, 0.92 and 0.82 for three sites in *FHL2*, and 0.92 and 0.94 for two sites in *GNPNAT1*.

### Validation of methylation changes in human pancreatic islets

We finally used pyrosequencing to technically validate and biologically replicate 7 CpG sites in *KLF14*, *FHL2* and *FAM123C* that exhibit significant age-related methylation changes in human islets (Fig. 5a–c). Technical validation was performed on islet DNA from 76 of the donors in the original 450 k array analysis, but the DNA was bisulfite treated on a different occasion. Importantly, the methylation data generated with the two methods correlated significantly for all analysed sites (Supplementary Data 11). To biologically replicate our methylation data, we analysed methylation of the same 7 CpG sites in islet samples from 38 donors, ages 19–72, not included in the array analysis (Supplementary Data 12). Importantly, we could replicate the significant findings on all 7 analysed sites (Supplementary Fig. 9a–c). In addition, we compared methylation of the 7 sites in islet donors younger than 40 (*n*=6) and older than 65 (*n*=10) years in the validation cohort. This revealed significantly increased methylation in elderly donors for all sites (Supplementary Fig. 9d). These data together with our previously published data^3^,^9^,^18^,^35^,^36^ support that the genome-wide analysis of DNA methylation in islets is robust and that the specificity of the array analysis is high.

## Discussion

Our study shows that aging associates with specific changes of DNA methylation in human pancreatic islets. These include loci in genes previously associated with diabetes risk and islet function. Importantly, we show that some age-related DNA methylation changes in pancreatic islets were similar in blood, suggesting that DNA methylation in blood may be used as a biomarker of DNA methylation in human islets. In addition, some of these blood-based epigenetic markers correlated with current and future insulin levels measured *in vivo* and were associated with lower risk of future T2D.

As the possibilities to monitor pancreatic islet function *in vivo* are limited, blood-based biomarkers for pancreatic islet function could have great clinical utility. We show that almost 60% of age-associated changes in DNA methylation in human islets occur also in blood. Our functional experiments further show that altered expression of some of these genes affects insulin secretion. For example, we show that methylation of *FHL2* associates with age, both in human islets and blood. In addition, the methylation level correlated with *FHL2* expression in islets and silencing *Fhl2* decreased insulin secretion *in vitro*. Furthermore, methylation of *FHL2* in blood was associated with lower T2D risk. Additional genes showing increased methylation in both pancreatic islets and blood during aging included *KLF14*, *FAM123C* and *ZNF518B*. *KLF14* encodes a transcriptional regulator and polymorphisms within this gene have been associated with T2D^37^. *FAM123C* encodes an activator of Wnt signalling^38^, a pathway important for β-cells^39^. Two recent studies have shown that *ZNF518B* encodes a protein that regulates histone methyltransferases^40^ and that a SNP in *ZNF518B* associates with blood glucose in patients with gout^41^. The identified associations between DNA methylation and future insulin secretion or T2D risk demonstrate, for example, that 1% higher DNA methylation of cg08097417 in *KLF14* is associated with 47.3 mU l^−1^ higher insulin secretion during an OGTT ∼10 years later, while 1% higher DNA methylation of cg16764848 in *GNPNAT1* is associated with an 8% lower risk for diabetes ∼11 years later. It should be noted that these analyses were adjusted for age, sex, BMI, HbA1c and family status.

DNA methylation was initially thought to be a silencing mark where increased methylation results in decreased expression^23^. However, emerging data show that the effect of DNA methylation depends on the genomic location and methylation may also affect transcriptional elongation, transcription of non-coding RNAs, alternative splicing and genomic stability^23^. While methylation of some of our identified sites in, for example, *FHL2*, *ZNF518B*, *GNPNAT1* and *HLTF* was associated with altered expression, the other identified CpG sites may affect other biological processes.

Interestingly, DNA methylation of all significant sites in human islets increased with age. This result is in line with age-associated methylation changes of sites found in other human tissues where methylation of the majority of sites increased with age^16^,^18^,^42^. On the other hand, increased age was found to decrease the global methylation level in human blood, several rodent tissues and cultured fibroblasts^15^,^43^,^44^. Although tissue-specific methylome alterations take place, methylation of genes such as *FHL2*, *KLF14*, *ELOVL2* and *GLRA1* seems to change in multiple tissues with age. The different mechanisms behind the tissue specific and more general methylation changes during a lifetime remain largely unknown. However, whether cells are replaced (for example, most blood cells) or remain throughout life may affect the impact of age on the methylome^45^. Age-associated effects on the activity of methyltransferases in different tissues may also contribute to alterations in the methylome.

As it has been proposed that CpG sites close to each other show a similar degree of methylation within a tissue and individual^30^, it is worth mentioning that 37 of the identified genes in our study had more than 1 site with age-associated changes in methylation. In addition, when we used pyrosequencing to analyse methylation of sites in *FHL2*, *KLF14*, *FAM123C*, *ZNF518B* and *GNPNAT1*, several surrounding sites showed altered methylation with age, suggesting that methylation in genomic regions rather than individual sites change with age.

To investigate if genes with age-associated methylation changes identified in pancreatic islets may contribute to altered insulin secretion^1^, we analysed the effects of *Fhl2*, *Zfp518b, Gnpnat1* and *Hltf* knockdown in clonal β-cells. While knockdown of *Zfp518b* and *Gnpnat1* mimics the age-associated decline in expression seen in human islets, knockdown of *Fhl2* and *Hltf* represents expression changes in the opposite direction of that seen in aging islets. Interestingly, all four genes affected insulin secretion. Of note, our functional experiments in β-cells suggest that the age-associated changes in methylation of *FHL2*, *ZNF518B* and *GNPNAT1* in human islets may be protective and compensatory changes to cope with increased demands of insulin due to insulin resistance in peripheral tissues. Conversely, age-associated changes in methylation and expression of *HLTF* may increase the risk of T2D. It is noteworthy that FHL2 through its LIM domains interacts with more than 50 partners involved in many signalling pathways^46^. Thus, changing FHL2 expression could affect different intracellular pathways and overexpression of Fhl2 in β-cells, that is, replicating the changes associated with aging, may therefore also reduce insulin secretion. *GNPNAT1* encodes an enzyme involved in N-linked glycosylation which is likely important for normal cellular function. HLTF is believed to have both DNA helicase and ubiquitinylating properties. To our knowledge, this is the first time these genes have been implicated in pancreatic β-cell function. It should be noted that these functional experiments were performed in a rodent β-cell line and differences between rodent and human β-cells may affect our results^47^. We did try to perform functional experiments in a human β-cell line (EndoC-βH1), but transfection with a control siRNA diminished the glucose-stimulated insulin secretion to levels where interpretation of the effects of knockdown was not feasible. We also wanted to investigate the effects of *KLF14* and *FAM123C* knockdown in β-cells. However, although both *KLF14* and *FAM123C* were clearly expressed in human islets and EndoC-βH1, the expression of these two genes was too low for reliable detection in the rat β-cell line. Hence, it was impossible to silence these two genes in rat β-cells.

Aging is a risk factor for T2D, a disease characterised by chronic hyperglycaemia resulting from impaired insulin secretion in combination with peripheral insulin resistance. Previous studies suggest that before insulin secretion fails and diabetes manifests, β-cells in elderly subjects try to compensate for insulin resistance with increased insulin secretion to prevent disease^1^. In our study, age was associated with elevated HbA1c in donors of pancreatic islets and participants in the Danish Family Study. Of note, both cohorts include non-diabetic individuals with a wide age span. Moreover, *in vivo* insulin secretion in absolute terms was elevated in elderly individuals of the Danish Family Study. However, we found no significant association between age and glucose-stimulated insulin secretion in human pancreatic islets cultured *in vitro*. This suggests that β-cells in islets from these elderly subjects are still able to respond to elevated glucose with increased insulin secretion. However, eventually aging β-cells may fail to adapt to the increasing demands for insulin production and T2D will develop. Interestingly, our functional experiments suggest that several age-associated epigenetic modifications and corresponding expression changes may compensate for the increasing demands for insulin during aging. Indeed, epigenetic changes in three out of four investigated genes in clonal β-cells seem to have compensatory and protective roles in the human islets. In support of this hypothesis, age-associated methylation changes of *KLF14* and *ZNF518B* in blood correlated with both current and future insulin levels measured *in vivo*. In addition, methylation of *KLF14*, *FHL2* and *GNPNAT1* was associated with lower diabetes risk in the Botnia prospective study. Nevertheless, this does not exclude an age-related β-cell defect in islets as deficient insulin secretion in elderly individuals is not always evident when analysed in absolute numbers, but rather when analysed in relation to insulin resistance^1^. In line, aging was associated with decreased DI in the Danish Family Study. In addition, a recent study found that age-related epigenetic changes in β-cells of old mice correlate with increased insulin secretion^48^.

We and others have previously published data from case–control studies, where we identified differential DNA methylation of numerous genes in islets from subjects with T2D versus controls^3^,^4^,^5^,^6^,^7^. However, in contrast to the present study where we found increased methylation of 241 sites with aging, diabetes was associated with decreased methylation of the majority of the significant sites. Also there was only a minor overlap between the genes identified in the present study and previous islet case–control cohorts, suggesting that different biological pathways underlie age- and diabetes-related DNA methylation changes. It is also worth mentioning that many of the hypomethylated sites identified in diabetic islets are located in intergenic regions^3^, while a large part of hypermethylation identified in aging islets take place in promoter regions. The results in our different studies are in line with data presented in a review by Jones *et al.*^49^, showing that methylation of CpG sites that are located in CpG islands and promoter regions generally increase with age while the methylation of intergenic CpG sites often decrease with age.

It is possible that aging is associated with changes in islet-cell composition and that this could affect our results^50^. However, we used electron microscopy to analyse the cellular composition of pancreatic islets and found no significant impact of age on β- or α-cell content. Another confounding factor could be an age-related increase in inflammatory cells in the islets, but comparison with a publicly available data set on human pancreatic islets^51^ showed that the expression of only 2 (*JAG2* and *IGFBP2*) out of 51 inflammation-related genes expressed in islets were nominally associated with age in our data set. This result supports that inflammation is not a prominent feature in islets from elderly subjects of our cohort. Also, a large number of the 241 identified CpG sites remained significant if the analysis was limited to donors with an age span of ∼20 years, making it unlikely that our identified age-associated methylation changes are due to altered islet-cell composition or inflammation. Also methylation data from blood samples may be affected by changes in cellular composition as aging has been found to associate with such changes^31^,^32^. However, data from the study by Reinius *et al.*^33^ presented here, together with a recent study by Yuan *et al*.^52^ that shows that most of the age-associated epigenetic drift seen in blood is independent of changes in blood cell composition, suggest that our methylation results are not caused by changes in cell composition. These data support that the age-associated epigenetic changes we identified in human islets and blood are unlikely to be influenced by altered cell composition.

Notably, the age-associated changes we observed in DNA methylation in the human islets are of quite large magnitude compared with data from some previous studies related to metabolic disease^4^,^5^,^53^. For example, absolute differences in DNA methylation larger than 20% between young and old donors were found for many sites in the human islets. Also, the identified differences in islet methylation between young and old donors were comparable to previously published results in blood^15^,^54^. Moreover, the impact of DNA methylation on future risk of diabetes was of a similar magnitude to some of the top T2D SNPs identified by GWAS^55^.

The interest in dissecting the impact of epigenetic variation on human diseases has increased over the last decade. However, for numerous diseases and epidemiological studies only blood is available and it is not always possible to get access to the pathogenic tissue of interest from humans^56^,^57^. Hence, it is important to understand if epigenetic variation in human blood cells mirrors epigenetic patterns in tissues central to the pathogenesis of disease. Indeed, our study demonstrates the value of blood for analysis of DNA methylation in relation to disease since we were able to replicate our age-related epigenetic findings from human islets in blood from large published studies^10^,^12^,^13^,^15^ and our own cohorts. It would be interesting to also replicate these findings in blood from the islet donors. However, we do not have access to other tissues from the included donors. Nevertheless, methylation data analysed in blood and pancreas from the same donors in the study by Slieker *et al.*^30^ support our findings.

We conclude that age-related epigenetic alterations in blood partially reflect DNA methylation changes in human pancreatic islets and associate with increased insulin secretion *in vivo*, as well as lower diabetes risk.

## Methods

### Human cohorts

Human pancreatic islets from 87 non-diabetic donors with a broad range in age (26–74 years) and HbA1c <6.5%, as determined with the mono S method, were obtained from the Nordic Network for Islet Transplantation at Uppsala University, Sweden. This pancreatic islet cohort is collected by the human tissue laboratory at Lund University Diabetes Centre and it is previously described^3^,^9^,^35^. Characteristics of these donors are given in Table 1. Informed consent for organ donation for medical research was obtained from pancreatic donors or their relatives in accordance with the approval by the regional ethics committees in Lund and Uppsala, Sweden.

Blood samples were obtained at two time points separated by ∼10 years (baseline and follow-up) at the fasted state from 112 individuals with a broad range in age (20–73 years at baseline and 32–83 years at follow-up), an HbA1c <6.5% at follow-up and without known disease (Supplementary Data 9). These individuals are part of a Danish Family Study previously described^26^,^27^, and includes 42 families with genetic risk for T2D. Insulin and C-peptide levels were measured in blood samples taken during fasting and a 75 g 2 h OGTT at both baseline and follow-up. DI was calculated as (100 × 0.144 × insulin OGTT_T30_)/(glucose OGTT_T30_ × (glucose OGTT_T30_–3.89)) × (10,000/square root of: 6.72 × (glucose OGTT_T0_ × insulin OGTT_T0_ × (glucose OGTT_T0_+glucose OGTT_T30_+glucose OGTT_T60_+glucose OGTT_T90_+glucose OGTT_T120_)/5 × (insulin OGTT_T0_+insulin OGTT_T30_+insulin OGTT_T60_+insulin OGTT_T90_+insulin OGTT_T120_)/5). Written informed consent was obtained from all participants and the research protocol was approved by the local Ethics Committee.

Blood samples were also obtained at baseline in the fasted state from 299 individuals of the Botnia prospective study^34^. This study includes individuals, which were healthy at baseline and they were followed prospectively with repeated OGTTs to detect progression to overt T2D. Here we included 105 participants who converted to T2D (converters) and 194 non-converters (controls) who remained healthy after 10.8±6.2 (mean±s.d.) years follow-up (Supplementary Data 10).

### Sample processing of human islets

Human islets were prepared by collagenase digestion and density gradient purification. Prior to nucleic acid purification, islets were cultured for 4.1±0.2 days at 37° (5% CO_2_) in CMRL 1066 culture medium (ICN Biomedicals, Costa Mesa, CA, USA) supplemented with 10 mM HEPES, 2 mM L-glutamine, 0.1 mM gentamicin, 0.27 μM amphotericin B (Fungizone, Thermo Scientific, Waltham, USA), 20 μg ml^−1^ Ciprofloxacin (Bayer HealthCare, Leverkusen, Germany) and 10 mM nicotinamide^58^. The purity of the islet preparations was 69.1±18.4% (mean±s.d.), as determined by dithizone staining^58^. There was no significant impact of age on purity of the islet preparations (Supplementary Fig. 10). Glucose-stimulated insulin secretion (stimulation index) from the human islets was measured in response to 1.67 and 16.7 mM glucose during glucose perfusion. Insulin content in the effluent collected in 6-min intervals was measured by enzyme immune assay specific for human insulin (Mercodia, Uppsala, Sweden). The stimulation index is calculated as the ratio between the area under the curve calculated for high glucose, divided by the area under the curve calculated for low glucose^59^. DNA and RNA were extracted from human pancreatic islets using the AllPrep DNA/RNA kit (Qiagen, Hilding, Germany) according to the manufacturer's instructions. Nucleic acid purity and concentration were determined using a nanodrop (NanoDrop Technologies, Wilmington, DE, USA). All DNA samples had an A260/280 ratio of 1.8–2.1, whereas the 260/280 ratios for RNA were 1.9–2.2. The integrity and quality of the RNA were assessed using the Bioanalyzer (Agilent Technologies, Santa Clara, CA, USA) and RNA integrity number (RIN) values were between 8.6 and 10. Islet α- and β-cell contents were determined in electron micrographs^3^ in islets from 12 donors aged 32–68 years. Hand-picked islets where fixed in 2.5% glutaraldehyde in freshly prepared Millonig and post fixed in 1% osmium tetroxide before being dehydrated and embedded in AGAR 100 (Oxford Instruments Nordiska, Lidingö, Sweden) and cut into ultrathin sections. The sections were put on Cu-grids and contrasted using uranyl acetate and lead citrate. The islet containing sections were examined in a JEM 1230 electron microscope (JEOL-USA. Inc., MA). Micrographs were analysed for β-cell content with ImageJ and in-house software programmed in Matlab. α- and β-cells were distinguished by means of granular appearance. The insulin granules of β-cells have a dense core surrounded by a white hale while α-cells have small dense granules.

### Global DNA methylation analysis

Genome-wide DNA methylation analysis of human pancreatic islets was performed with the Infinium HumanMethylation450 BeadChip kit (Illumina, Inc., CA, USA). Genomic DNA (500 ng) was bisulfite converted using an EZ DNA methylation kit (Zymo Research, Orange, CA, USA) and then used to analyse DNA methylation with Infinium assay using the standard Infinium HD Assay Methylation Protocol Guide (part number 15019519, Illumina). Samples were randomly bisulfite treated and distributed on the arrays. The bead chips were imaged with the Illumina iScan. The Infinium HumanMethylation450 BeadChip contains 485,577 probes and covers 99% of all RefSeq genes with the capacity for 12 samples per chip^22^. The GenomeStudio methylation module software was used to calculate the raw methylation score for each DNA methylation site, which is represented as methylation *β* value. The *β* values are calculated as *β*=intensity of the methylated allele (*M*)/(intensity of the unmethylated allele (*U*)+intensity of the methylated allele (*M*)+100). All samples passed GenomeStudio quality control steps based on built-in control probes for staining, hybridization, extension and specificity and displayed high quality bisulfite conversion efficiency with an intensity signal above 4,000 (ref. 60). Probes were then filtered based on detection *P* value, and probes with a mean detection *P* value >0.01, as determined by the Illumina platform, were removed from further analysis. In total, DNA methylation data were obtained for 483,031 probes. Since the cohort included islets from both males and females, Y-chromosome data were removed and subsequently DNA methylation data from 482,954 probes remained for further analysis. *β* values were converted to *M* values for further analysis (*M*=log2(*β*/(1−*β*)) (ref. 61). Background correction and quantile normalization were performed using the lumi package from bioconductor^62^. To identify DNA methylation changes that associate with age, the methylation data were analysed using a linear regression model with the limma package in Bioconductor^63^,^64^ including bisulfite treatment, sex, BMI, purity of the islets, days in culture and HbA1c as covariates. A FDR analysis was performed to correct for multiple testing and *q* values<0.05 were considered significant. Since *β* values are easier to interpret biologically, *M* values were reconverted into *β* values and were then used when describing the data and when generating figures. Moreover, since some probes on Illumina's DNA methylation chip may cross-react to multiple locations in the genome, we used the published data by Chen *et al.*^65^ to evaluate the number of possible cross-reactive probes among our significant methylation data.

### Pyrosequencing

Pyrosequencing was used to analyse DNA methylation in whole blood from 112 individuals in the Danish Family Study. Selected and surrounding CpG sites in six genes were analysed; cg06639320, cg22454769 and cg24079702 in *FHL2*, cg08097417 and cg22285878 in *KLF14*, cg23606718 in *FAM123C*, cg16295725 and cg23995914 in *ZNF518B,* cg16764848 in *GNPNAT1,* and cg05555455 and cg18549036 in *HLTF*. Assays (Supplementary Data 13) were designed with the PyroMark Assay Design 2.0 software (Qiagen). Pyrosequencing was performed with the PyroMark Q96 ID system (Qiagen) and all procedures were performed according to the manufacturer's recommendations. In short, genomic DNA from whole blood was isolated with the QIAmpDNA Blood Mini Kit (Qiagen). 400 ng of the DNA were bisulfite converted with the EpiTect 96 Bisulfite Kit (Qiagen, all samples from the Danish Family Study) or the EZ DNA methylation kit (islet samples for technical validation and biological replication, and samples from the Botnia prospective study) and 10 ng of the bisulfite-converted DNA were used as input for each PCR reaction. The PyroMark PCR Master Mix kit (Qiagen), streptavidin-coated beads (GE Healthcare, Uppsala, Sweden), PyroMark Gold Q96 reagents (Qiagen), PyroMark Q96 Vacuum Workstation and PyroMark Q96 software (version 2.5.8, Qiagen) were used for the determination and analysis of DNA methylation, all according to the manufacturers' recommendations.

### mRNA expression analysis

mRNA expression was analysed with the Affymetrix GeneChip Human Gene 1.0 ST whole transcript based array (Affymetrix, Santa Clara, CA, USA) according to the manufacturer's recommendations. We computed robust multichip average expression measure using the oligo package from Bioconductor^66^.

### Transcription factor-binding site analysis

LASAGNA-Search 2.0 (ref. 67) was used to analyse which transcription factor-binding sites overlapped with the significant CpG sites in the promoters for *FHL2* and *HLTF*. Sequences of DNA stretching from 5 bp upstream of the first CpG site to 5 bp downstream of the last CpG site were entered into the search tool. TRANSFAC matrices and aligned models were used and the significance cut-off was set to the default value.

### Luciferase assays

The protocol has been described in detail elsewhere^6^. Briefly, a 2,000 bp fragment of the *ZNF518B* and *GNPNAT1* promoters immediately upstream of the transcription start site, covering CpG sites with age-associated changes in methylation was cloned into a CpG-free luciferase reporter vector (pCpGL-basic) kindly provided by Dr Klug and Dr Rehli^68^. Cloning of sequences and insertion into the pCpGL-basic vector was performed by GenScript (GenScript USA Inc., Piscataway, NJ, USA). Two different DNA methyltransferases, SssI and HhaI (2.5 U per μg DNA) (New England Biolabs, Frankfurt, Germany), were then used to methylate the constructs. SssI methylates all cytosine residues within the double-stranded dinucleotide recognition sequence CG and HhaI methylates only the internal cytosine residue in GCGC sequences. The clonal rat β-cell line 832/13 INS-1 (ref. 69), a kind gift from Dr Christopher Newgard at Duke University, was co-transfected with 25 ng methylated or mock-methylated pCpGL-vector including either of the two promoter inserts together with 4 ng of pRL renilla luciferase control reporter vector (Promega, Madison, WI, USA). Firefly and renilla luciferase luminescence, as a value of transcriptional activity, was measured for each construct with the Dual-Glo Luciferase Assay System (Promega) and an Infinite M200 PRO multiplate reader (Tecan Group Ltd., Männedorf, Switzerland). Cells transfected with an empty pCpGL-vector were used as a background control for firefly luciferase results, and untransfected cells were used as a background for renilla results.

### Cell culture and siRNA-mediated knockdown

For transfection, 2.0 × 10^5^ 832/13 INS-1 β-cells per well were seeded on 24-well plates and cultured overnight. Cells were then transfected with 25 nM siRNA (Life Technologies, Paisley, UK) by using Dharmafect I (Thermo Scientific) according to the manufacturer's instructions. The siRNAs used were s133583 (siFhl2), s176980 (siGnpnat1), s149330 (siHltf), s176846 (siZfp518b) and a negative control siRNA (5′-GAGACCCUAUCCGUGAUUAUU-3′). RNA was isolated with the GeneJET RNA purification kit (Thermo Scientific) 72 h post transfection and converted to complementary DNA (cDNA) with the RevertAid First Strand cDNA synthesis kit (Thermo Scientific). Knockdown was verified on a ViiA7 qPCR system (Life Technologies) with TaqMan assays (Life Technologies) for *Fhl2* (Rn00581565_m1), *Gnpnat1* (Rn01414655_m1) and *Hltf* (Rn01493406_m1). Assays for *Hprt1* (Rn01527840_m1) and *Ppia* (Rn00690933_m1) were used as endogenous controls. Expression of *Zfp518b* was analysed with SybrGreen (Thermo Scientific) and the following primers; 5′-CGTCCGGGCCTGTTGG-3′ and 5′-AGGCCCAGAAGTCAAGTGTG-3′ and normalized to the expression of *Hprt1* (5′-CCCAGCGTCGTGATTAGTGA-3′ and 5′-TGGCCTCCCATCTCCTTCAT-3′) and *Ppia* (5′-AGGATTCATGTGCCAGGGTG-3′ and 5′-CTCAGTCTTGGCAGTGCAGA-3′). Quantification was done with the ΔΔCt method.

### Insulin secretion in clonal β-cells

Insulin secretion was determined in 1 h static incubations 72 h post transfection. Transfected β-cells were washed in HEPES balanced salt solution (HBSS; 114 mM NaCl, 4.7 mM KCl, 1.2 mM KH_2_PO_4_, 1.16 mM MgSO_4_, 20 mM HEPES, 2.5 mM CaCl_2_, 25.5 mM NaHCO_3_, 0.2% bovine serum albumin, pH 7.2) supplemented with 2.8 mM glucose, followed by a 2 h pre-incubation at 37 °C in the same buffer. Insulin secretion was then determined by stimulating the cells with fresh HBSS with 2.8 or 16.7 mM glucose for 1 h. Insulin released into the buffer was measured with a rat insulin ELISA (Mercodia) and normalized to the total amount of protein in each well as measured with the BCA Protein Assay Kit (Thermo Scientific).

### Statistics

The association between age and methylation in pancreatic islets was analysed with a linear regression model using methylation *M* values with the limma package in bioconductor^63^,^64^. Sex BMI, HbA1c, bisulfite treatment, days in culture and islet purity were included as covariates in the analysis. FDR analysis was performed to correct for multiple testing^70^. Linear regression models were used to test associations between age and HbA1c, BMI or glucose-stimulated insulin secretion including the same covariates as above except for days in culture, islet purity, HbA1c or BMI when appropriate. Correlation between age and islet-cell composition was analysed with Spearman correlation. The genomic distribution of significant sites was analysed with a *χ*^2^-test. Associations between age and DNA methylation measured by pyrosequencing were analysed with linear mixed effects models for the Danish Family Study. Here age, sex, BMI and HbA1c were included as fixed factors, whereas family number/pedigree was included as a random factor in all models. Incident T2D was analysed with a Cox regression model. Data were treated as left truncated and right censored. The covariate measurements were made at entry time. All regression analyses were performed with a robust variance estimate to adjust for within-family dependence. Correlation between *β* values of DNA methylation and gene expression was analysed with Spearman's rank test. Functional studies were analysed with Mann–Whitney test or Wilcoxon signed rank test as indicated. Statistical analyses were performed with R (http://www.r-project.org/) and Statistical Package for the Social Sciences (SPSS, IBM, Armonk, NY, USA).

## Additional information

**Accession codes:** The methylation and expression array data are available at http://www.ncbi.nlm.nih.gov/geo/ with the accession numbers GSE62640 and GSE54279, respectively.

**How to cite this article:** Bacos, K. *et al.* Blood-based biomarkers of age-associated epigenetic changes in human islets associate with insulin secretion and diabetes. *Nat. Commun.* 7:11089 doi: 10.1038/ncomms11089 (2016).

## Supplementary Material

Supplementary Information Supplementary Figures 1-10

Supplementary Data 1 Methylation sites that are significantly associated with age in human pancreatic islets

Supplementary Data 2 Cross-reactive probes

Supplementary Data 3 Methylation sites that were significantly associated with age in human pancreatic islets in a narrower age-segment (donors within mean age ± 1 SD)

Supplementary Data 4 Literature search on genes with age-associated changes in methylation in human pancreatic islets

Supplementary Data 5 DNA methylation and gene expression correlates in human pancreatic islets

Supplementary Data 6 Transcriptional repressors that bind to FHL2 and HLTF promoter sequences containing CpG sites that are significantly associated with age

Supplementary Data 7 The transcriptional repressor NRSF binds to a sequence in the GNPNAT1 promoter methylated by HhaI.

Supplementary Data 8 Methylation sites that are significantly associated with age in both pancreatic islets and blood

Supplementary Data 9 Clinical characteristics of the 112 participants from the Danish Family Study

Supplementary Data 10 Clinical characteristics of participants from the Botnia prospective study

Supplementary Data 11 Technical validation of islet DNA methylation data

Supplementary Data 12 Characteristics of the 38 islet donors in the biological replication cohort

Supplementary Data 13 Primers used for pyrosequencing analysis

## Figures and Tables

**Table 1:** Characteristics of non-diabetic pancreatic islet donors.

**Phenotype**	**Average**	**Min–max**
*n*=87 (53 M, 34 F)		
Age (years)	56.7±10.5	26–74
BMI (kg m^−2^)	25.8±3.4	17.6–40.1
HbA1c* (%)	5.6±0.4	4.3–6.4

BMI, body mass index; F, female; M, male.

Data are presented as mean±s.d.

^*^Data available for 74 donors.

**Table 2:** Associations between age and clinical parameters in the Danish Family Study at follow-up.

**Phenotype**	**Regression coefficient**	***P*** **value**
HbA1c (%)	0.014	1.5 × 10^−7^
Fasting glucose (mM)	0.0086	0.016
120** **min glucose	0.045	8.8 × 10^−4^
Fasting insulin (pM)	0.17	0.51
120** **min insulin	6.35	0.0092
Fasting C-peptide (pM)	9.44	1.4 × 10^−5^
120** **min C-peptide	52.28	3.3 × 10^−8^
Disposition index	−14.2	0.036

BMI, body mass index.

The association analyses were adjusted for sex, body mass index (BMI) and family.

**Table 3:** Associations (*P*<0.05) between DNA methylation of CpG sites in *KLF14* and *ZNF518B* and measurements of insulin and C-peptide levels in the Danish Family Study at baseline.

**CpG site**	**Phenotype**	**Regression coefficient**	***P*** **value**
cg08097417 (*KLF14*)	120 min insulin (mU l^−1^)	24.4	0.019
	120 min C-peptide (nM)	106.2	0.014
cg22285878 (*KLF14*)	Fasting C-peptide	13.3	0.047
	120 min C-peptide	79.7	0.016
CpG 3 (*KLF14*)	120 min insulin	17.6	0.038
CpG 1 (*ZNF518B*)	120 min insulin	21.3	0.027
CpG 3 (*ZNF518B*)	Fasting insulin	2.0	0.031

The association analyses were adjusted for sex, body mass index (BMI) and family.

**Table 4:** Associations (*P*<0.05) between DNA methylation of CpG sites in *KLF14* and *ZNF518B* at baseline, and measurements of insulin and C-peptide levels at follow-up in the Danish Family Study.

**CpG site**	**Phenotype**	**Regression coefficient**	***P*** **value**
cg08097417 (*KLF14*)	120 min insulin (mU l^−1^)	47.3	0.0049
	120 min C-peptide (nM)	142.4	0.030
cg22285878 (*KLF14*)	120 min insulin	30.4	0.020
	120 min C-peptide	109.9	0.031
CpG 2 (*KLF14*)	120 min insulin	59.1	0.0029
cg23995914 (*ZNF518B*)	Fasting C-peptide	−25.6	0.014
CpG 1 (*ZNF518B*)	Fasting insulin	3.7	0.042
	120 min insulin	29.4	0.045
CpG 2 (*ZNF518B*)	Fasting C-peptide	−27.1	0.030

The association analyses were adjusted for sex, body mass index (BMI) and family.

**Table 5:** Associations (*P*<0.05) between DNA methylation of CpG sites in *KLF14, FHL2* and *GNPNAT1,* and lower risk for future T2D in the Botnia prospective cohort.

**CpG site**	**Hazard-ratio (95% CI)**	***P*** **value**
CpG 1 (*KLF14*)	0.88 (0.78-0.99)	0.03
CpG 2 (*FHL2*)	0.94 (0.88-0.99)	0.037
CpG 3 (*FHL2*)	0.92 (0.84-0.99)	0.049
CpG 5 (*FHL2*)	0.82 (0.68-0.99)	0.037
cg16764848 (*GNPNAT1*)	0.92 (0.88-0.96)	8.0 × 10^−5^
CpG 1 (*GNPNAT1*)	0.94 (0.90-0.98)	0.003

CI, confidence interval.

The association analyses were adjusted for age, sex, body mass index (BMI), HbA1c at baseline and family.
