# Supplementary material for: Blood-based biomarkers of age-associated epigenetic changes in human islets associate with insulin secretion and diabetes
Source: Nat Commun. 2016 Mar 31;7:11089. doi: 10.1038/ncomms11089 (PMC4821875; doi:10.1038/ncomms11089)
Supplement: Supplementary Information — Supplementary Figures 1-10 [file ncomms11089-s1.pdf]

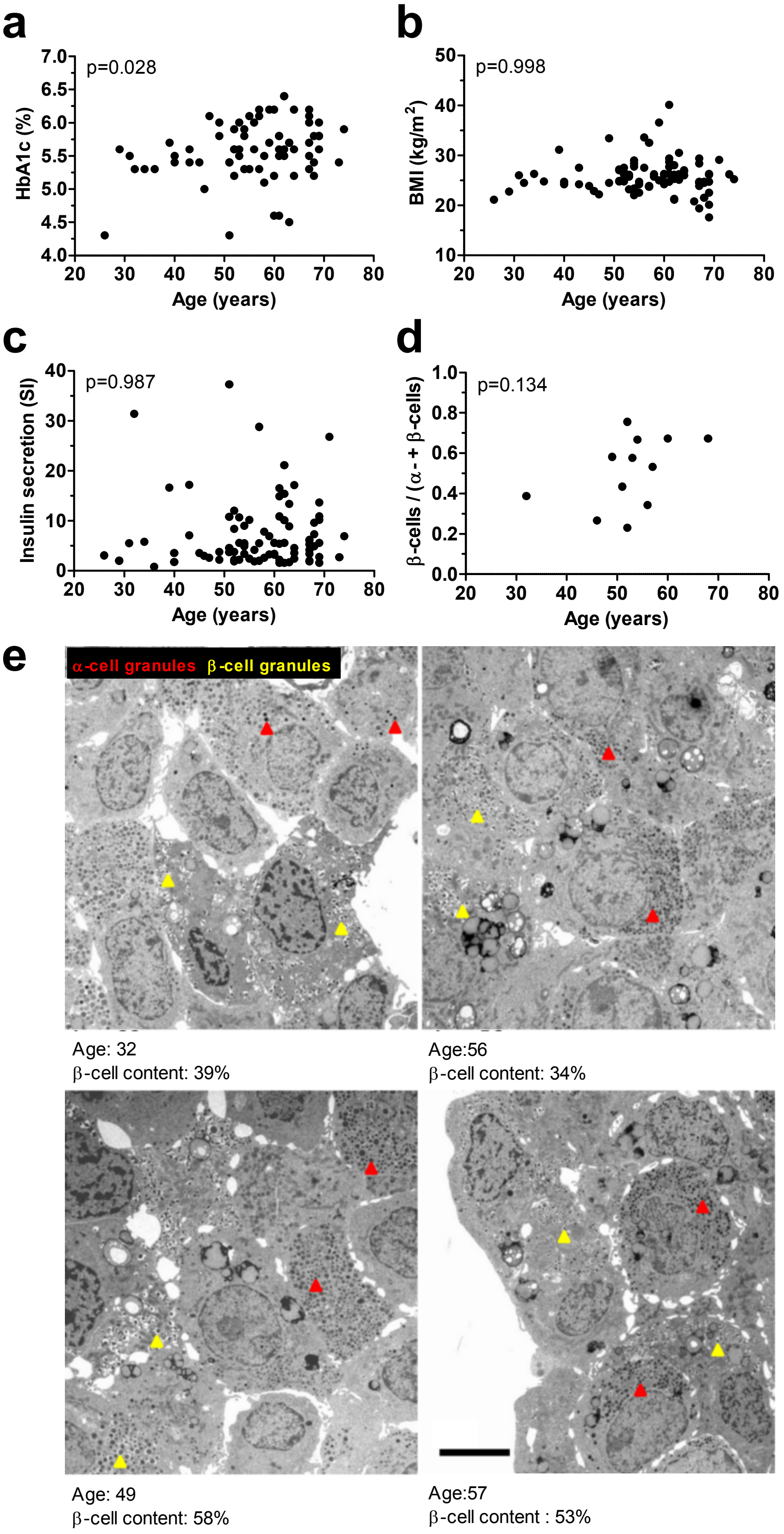

Supplementary Figure 1. Age correlates positively with HbA1c levels. Correlation between age and HbA1c levels (a), BMI (b), insulin secretion (c), and islet β-cell content (d) in pancreatic islet donors as analysed by linear regression analysis (a-c, n=87) or Spearman correlation (d, n=12). (e) Representative electron micrographs of pancreatic islets from four donors, ages 32-57. β- and α-cell granules are indicated by red and yellow arrowheads, respectively. Scale bar in the lower right panel is 5μM.

Supplementary Figure 1 - Ling

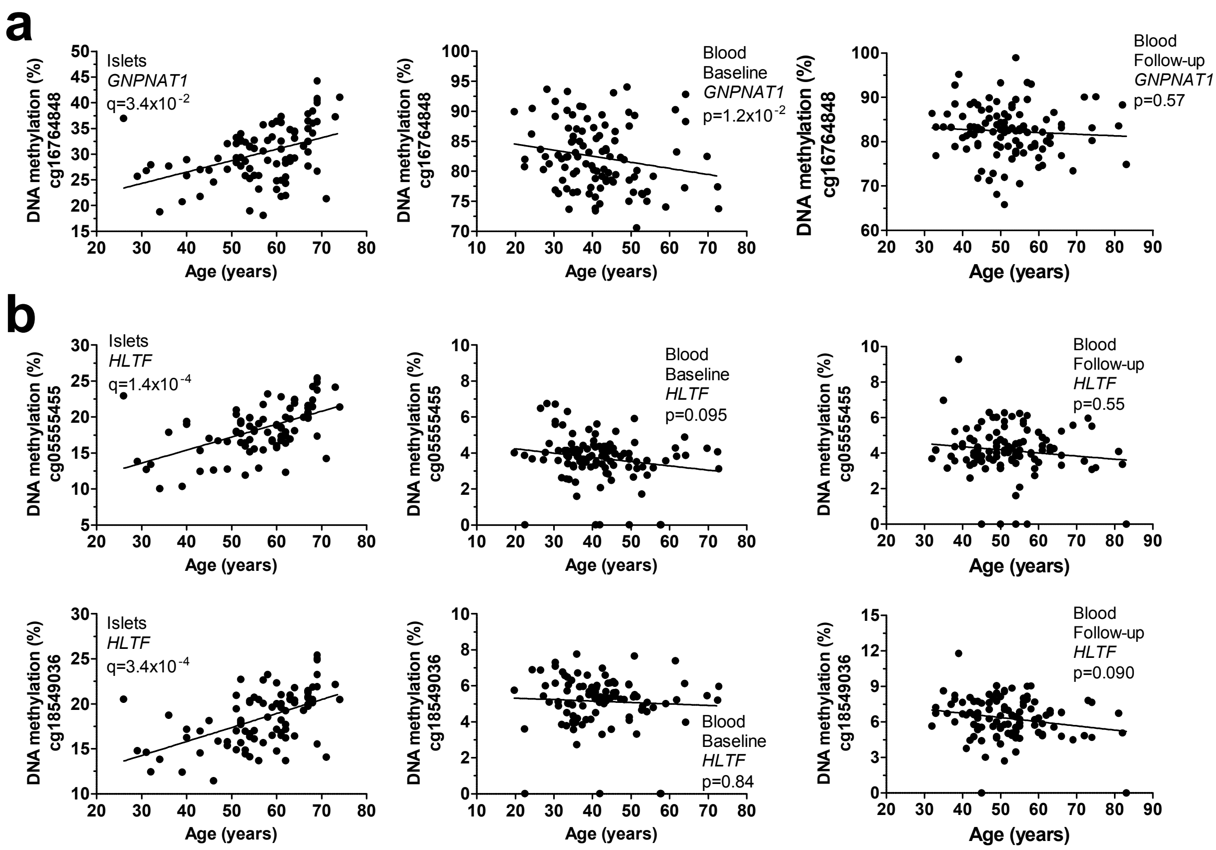

Supplementary Figure 2. Age-associated DNA methylation changes of *GNPNAT1* and *HLTF* in human pancreatic islets and blood. The association between age and DNA methylation of sites in *GNPNAT1* (a) and *HLTF* (b) in pancreatic islets from the human islet cohort (n=87, left panels), and blood from the Danish Family Study (n=112) at baseline (middle panels) and follow-up (right panels). Data were analysed by linear regression.

Supplementary Figure 2 - Ling

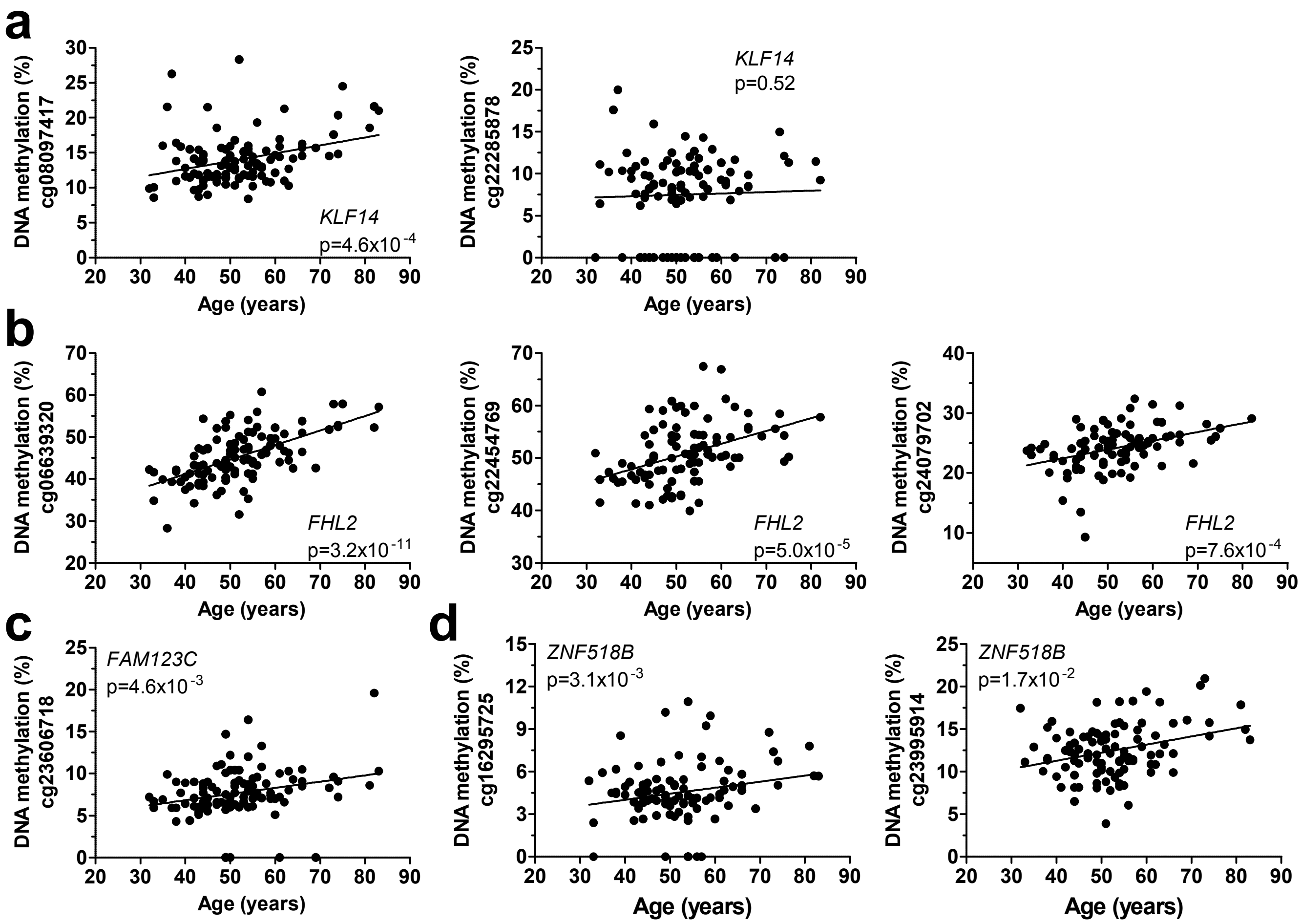

Supplementary Figure 3. DNA methylation of CpG sites in *KLF14*, *FHL2*, *FAM123C*, and *ZNF518B* at follow-up in blood from the Danish Family Study. Pyrosequencing of DNA from donors in the Danish Family Study revealed that methylation of CpG sites in *KLF14* (a), *FHL2* (b), *FAM123C* (c), and *ZNF518B* (d) associated significantly with age also at follow-up as analysed by linear regression (n=112).

Supplementary Figure 3 - Ling

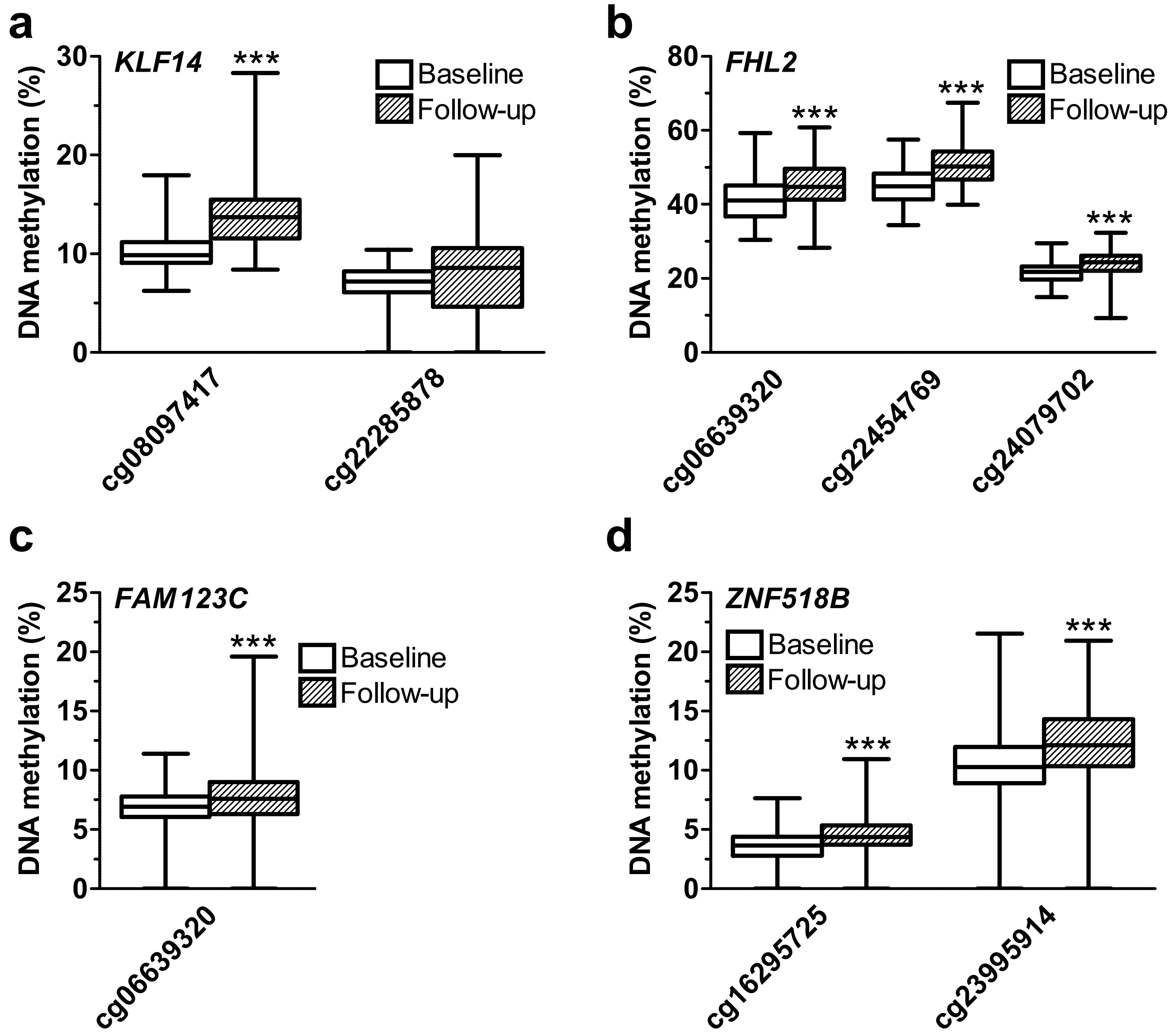

Supplementary Figure 4. DNA methylation of *KLF14*, *FHL2*, *FAM123C*, and *ZNF518B* in blood increased over time. The difference in methylation of indicated sites in blood samples from the Danish Family Study (n=112) at baseline and at follow-up was analysed with a Wilcoxon signed rank test. *** $p < 0.0001$.

Supplementary Figure 4 - Ling

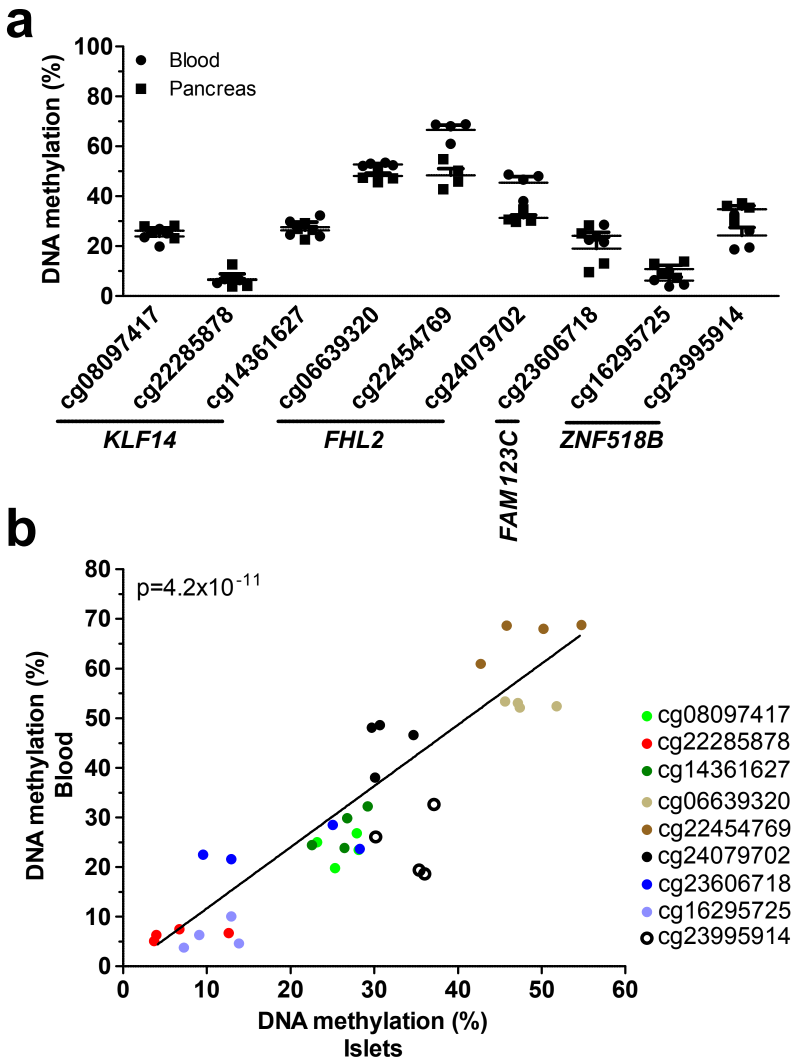

Supplementary Figure 5. DNA methylation of CpG sites in *KLF14*, *FHL2*, *FAM123C*, and *ZNF518B* are similar in pancreas and blood. (a) Analysis of published data by Slieker et al[30] show that methylation of the studied sites in *KLF14*, *FHL2*, *FAM123C*, and *ZNF518B* is similar in pancreas and blood taken from the same donors (n=4). *GNPNAT1* and *HLTF* were excluded from this analysis as they did not exhibit similar age-associated changes in DNA methylation in our islet and blood data. (b) Methylation of the nine sites in *KLF14*, *FHL2*, *FAM123C*, and *ZNF518B* in pancreas and blood taken form the same donors correlated significantly.

Supplementary Figure 5 - Ling

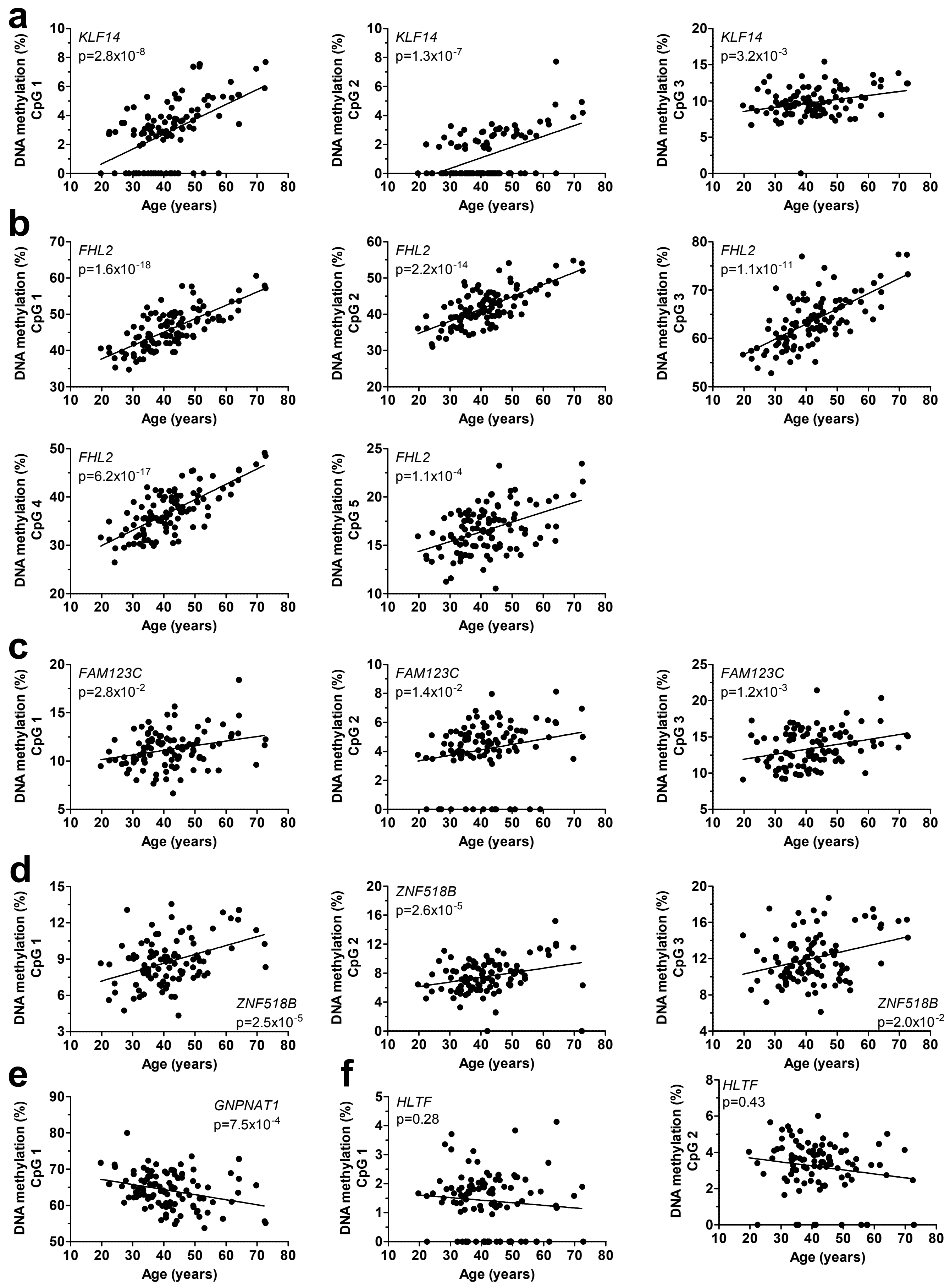

Supplementary Figure 6. DNA methylation of surrounding CpG sites in *KLF14*, *FHL2*, *FAM123C*, *ZNF518B* and *GNPNAT1* associates with age at baseline in the Danish Family Study. Pyrosequencing revealed that several surrounding sites in *KLF14* (a), *FHL2* (b), *FAM123C* (c), *ZNF518B* (d), and *GNPNAT1* (e) were significantly associated with age in blood at baseline (n=112). However, the surrounding sites in *HLTF* (f) did not associate with age at baseline. Data were analysed by linear regression.

Supplementary Figure 6 - Ling

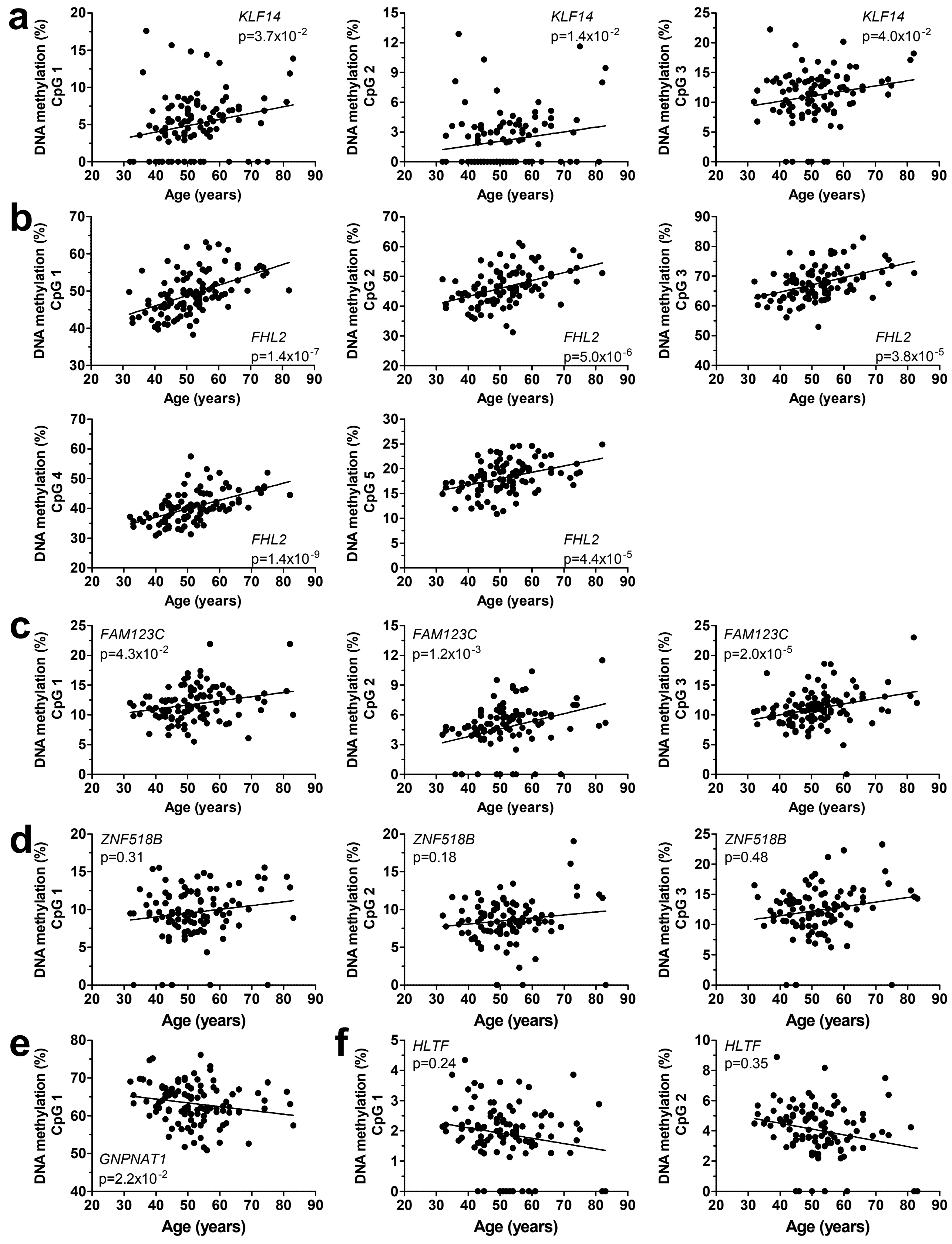

Supplementary Figure 7. DNA methylation of surrounding CpG sites in *KLF14*, *FHL2*, *FAM123C*, and *GNPNAT1* associates with age at follow-up in the Danish Family Study. Pyrosequencing revealed that several surrounding sites in *KLF14* (a), *FHL2* (b) *FAM123C* (c), and *GNPNAT1* (e) were significantly associated with age in blood at follow-up (n=112). However, the surrounding sites in *ZNF518B* (d) and *HLTF* (f) did not associate with age at follow-up. Data were analysed by linear regression.

Supplementary Figure 7 - Ling

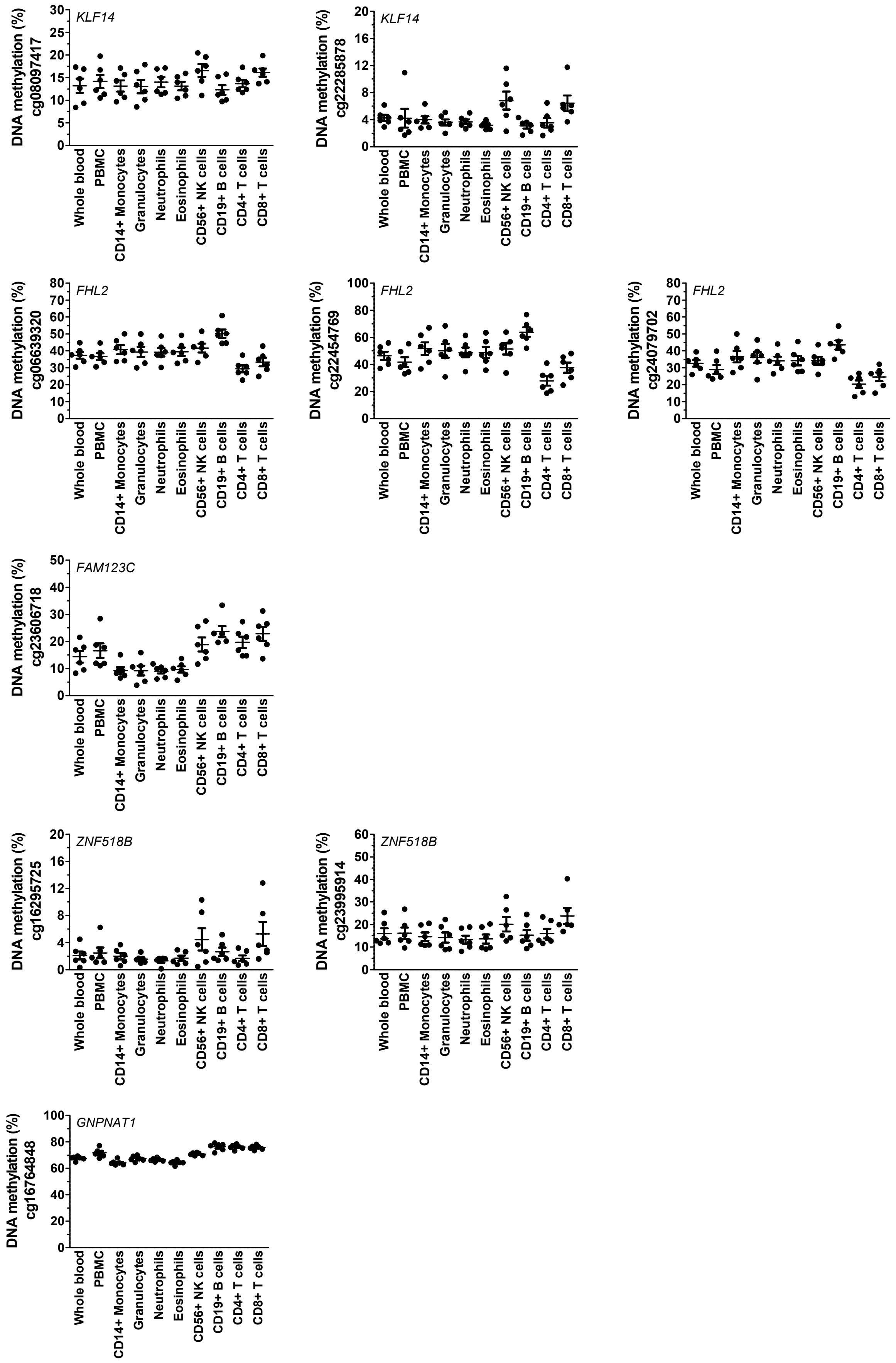

Supplementary Figure 8. DNA methylation of *KLF14*, *FHL2*, *FAM123C*, *ZNF518B*, and *GNPNAT1* in isolated human blood cell populations. Published methylation data by Reinius et al[33] from isolated blood cell populations suggest that the age-associated methylation changes identified in *KLF14*, *FHL2*, *FAM123C*, *ZNF518B*, and *GNPNAT1* are not due to an altered blood cell composition (n=6).

Supplementary Figure 8 - Ling

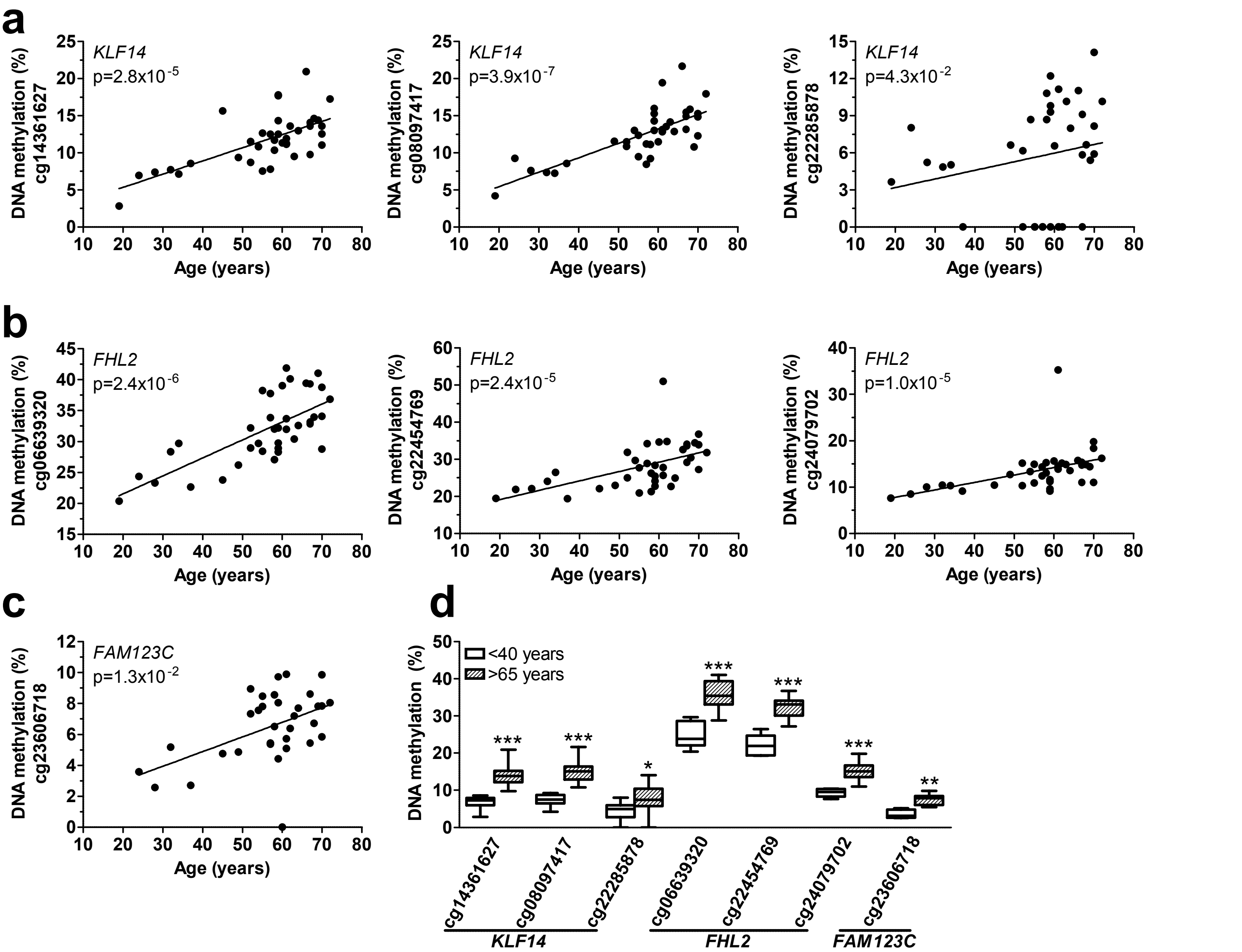

Supplementary Figure 9. Biological replication of age-associated DNA methylation changes in human pancreatic islets. Pyrosequencing analysis of methylation of seven age-associated CpG sites in *KLF14* (a), *FHL2* (b), and *FAM123C* (c) on DNA from pancreatic islets from a replication cohort of 38 donors verified that methylation of all analysed CpG sites correlated significantly with age. Data were analysed with Spearman correlations. (d) DNA methylation differed significantly for all seven sites in pancreatic islets from young (<40 years, n=6) versus old (>65 years, n=10) donors in the validation cohort. Data analysed with a Mann-Whitney U test. *p<0.05, **p<0.01, and ***p<0.001.

Supplementary Figure 9 - Ling

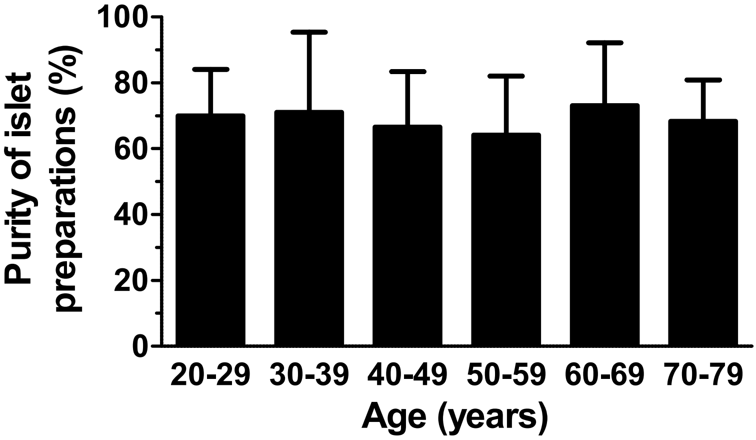

Supplementary Figure 10. There was no impact of age of the donor on the purity of islet preparations, thus excluding such a bias as confounding factor.

Supplementary Figure 10 - Ling
